# Epigenetically modified *AP-2α* by DNA methyltransferase facilitates glioma immune evasion by upregulating PD-L1 expression

**DOI:** 10.1038/s41419-023-05878-x

**Published:** 2023-06-17

**Authors:** Shengwen Long, Guixiang Huang, Mi Ouyang, Kai Xiao, Hao Zhou, Anyi Hou, Zhiwei Li, Zhe Zhong, Dongmei Zhong, Qinghao Wang, Shuanglin Xiang, Xiaofeng Ding

**Affiliations:** 1 The National & Local Joint Engineering Laboratory of Animal Peptide Drug Development, College of Life Science, Hunan Normal University, Changsha, 410081 China; 2 Key Laboratory of Model Animals and Stem Cell Biology in Hunan Province, School of Medicine, Hunan Normal University, Changsha, 410013 China; 3 Department of Neurosurgery, Xiangya Hospital of Central South University, Changsha, Hunan 410008 China; 4 Department of Neurosurgery, Hunan Provincial Tumor Hospital, The Affiliated Tumor Hospital of Xiangya Medical School of Central South University, Changsha, Hunan 410013 China

**Keywords:** Immunosurveillance, Tumour-suppressor proteins

## Abstract

Programmed death-ligand 1 (PD-L1) ensures that tumor cells escape T-cell-mediated tumor immune surveillance. However, gliomas are characteristic of the low immune response and high-resistance therapy, it is necessary to understand molecular regulatory mechanisms in glioblastoma, especially the limited regulation of PD-L1 expression. Herein, we show that low expression of AP-2α is correlated with high expression of PD-L1 in high-grade glioma tissues. AP-2α binds directly to the promoter of the *CD274* gene, not only inhibits the transcriptional activity of *PD-L1* but enhances endocytosis and degradation of PD-L1 proteins. Overexpression of AP-2α in gliomas enhances CD8^+^ T cell-mediated proliferation, effector cytokine secretion, and cytotoxicity in vitro. Tfap2a could increase the cytotoxic effect of Cd8^+^ T cells in CT26, B16F10, and GL261 tumor-immune models, improve anti-tumor immunity, and promote the efficacy of anti-PD-1 therapy. Finally, the EZH2/H3K27Me3/DNMT1 complex mediates the methylation modification of *AP-2α* gene and maintains low expression of AP-2α in gliomas. 5-Aza-dC (Decitabine) treatment combines with anti-PD-1 immunotherapy to efficiently suppress the progression of GL261 gliomas. Overall, these data support a mechanism of epigenetic modification of *AP-2α* that contributes to tumor immune evasion, and reactivation of AP-2α synergizes with anti-PD-1 antibodies to increase antitumor efficacy, which may be a broadly applicable strategy in solid tumors.

## Introduction

Blockade of the immune checkpoint has been a critical breakthrough in clinical cancer therapy [1, 2]. The interaction of PD-L1 and programmed death-1 (PD-1) inhibits T lymphocyte proliferation, suppresses CD8^+^ T cytotoxicity, and evades immune surveillance, leading to tumor progression [3]. Blocking the interaction between PD-1 and PD-L1 activates T cell responses to target tumor cells expressing PD-L1. Anti-PD-1 immunotherapy functions as an FDA-approved drug for lung cancer, bladder cancer, and melanoma [4]. The expression of PD-L1 proteins in cancer cells has been found as a biomarker predicting the clinical response of patients [5]. However, PD-L1 expression alone is not necessarily associated with the efficacy of immune checkpoint blockade [6]. Therefore, it is urgent to elucidate the complex mechanisms of *PD-L1* regulation to improve PD-1/PD-L1-based immunotherapies.

Gliomas originating from primary brain tumors display the most aggressive behavior and show a dismal prognosis. Temozolomide (TMZ), a first-line clinical drug for glioblastoma, could prolong overall survival in MGMT-negative patients [7]. Treatment options are limited, and the relapse rate is high in gliomas [8]. Several isolated reports showed that the PD-1 inhibitor, in combination with surgical therapy, enhances cytotoxic T cells in recurrent glioblastoma [9]. PD-1 antibody improved the immune response and prolonged the survival in recurrent gliomas [10]. Although immune checkpoint inhibitor trials in glioblastoma have been disappointing [11], blocking the PD-L1/PD-1 interaction may represent a potential treatment for gliomas.

The transcription factor AP-2α was downregulated in solid tumors and suppressed the malignant behaviors of tumor cells [12–17]. However, the detailed function of AP-2α in anti-tumor immunity has not been reported. AP-2α could induce the expression of *the TLR2* gene, which mediates innate and adaptive immune responses [18, 19]. AP-2α suppressed IFNGR1 expression and impaired IFN-γ signaling [20], suggesting that AP-2α might influence immune responses in cancer cells. And INF-γ upregulates the JAK-2/STAT1/IRF-1 signaling pathway and stimulates PD-L1 expression [21]. We speculated that AP-2α might correlate with the PD-L1/PD-1 pathway in tumor cells. Herein, we demonstrated the binding and negative regulatory correlation between AP-2α and the *PD-L1* promoter. Moreover, Tfap2a can enhance anti-tumor immunity and the efficacy of anti-PD-1 therapy. Finally, the sensitivity to the anti-PD-1 antibody is augmented by Decitabine via suppressing *AP-2α* methylation. Therefore, our results suggest a novel function of AP-2α in anti-tumor immunity in gliomas.

## Results

### In gliomas, low expression of AP-2α correlates with high expression of PD-L1

**Fig. 1:**
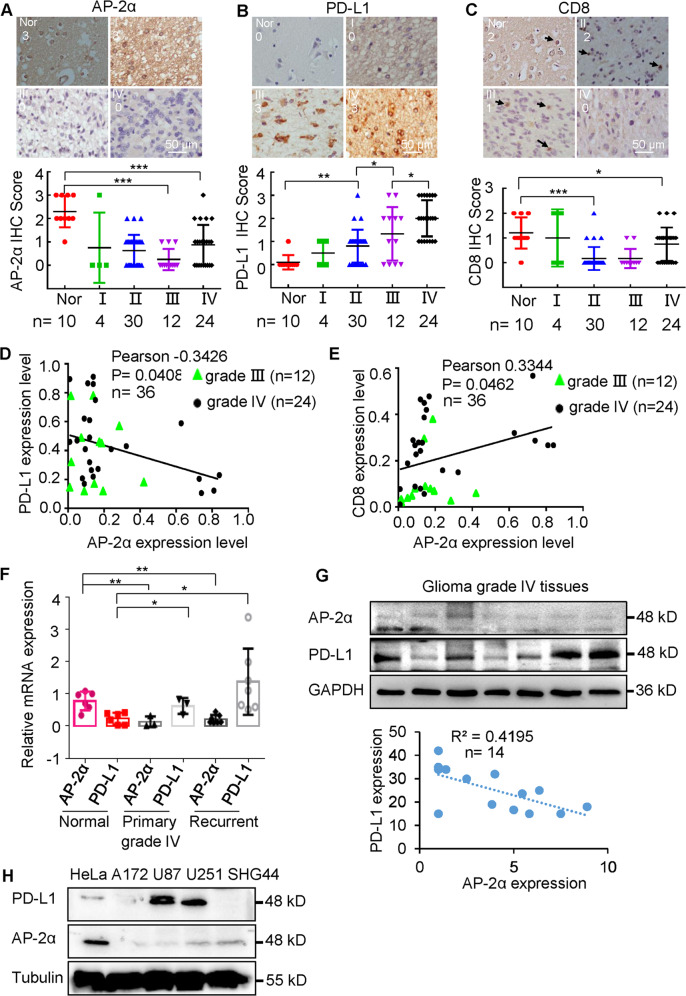
The negative correlation of AP-2α and PD-L1 expression in glioma tissues. **A**–**C** The expression of AP-2α, PD-L1, and CD8 in glioma tissues analyzed by IHC and the corresponding staining scores in different grades of gliomas. **D**, **E** The correlation between AP-2α/PD-L1 and AP-2α/CD8 expression in grade III/IV gliomas based on IHC scores. **F** qRT-PCR analysis of AP-2α and PD-L1 expression in glioma tissues. **G**, **H** Western blot analysis of protein expression of AP-2α and PD-L1 in glioma grade IV tissues and glioma cell lines.

We analyzed the expression of AP-2α and PD-L1 in gliomas by IHC staining. AP-2α was lowly expressed [14], while PD-L1 is highly expressed in high-grade gliomas (Fig. 1A, B and Fig. S1A), but the relationship between AP-2α and PD-L1 expression in gliomas is unclear. We showed the inverse correlation of AP-2α and PD-L1 expression in high-grade gliomas (Fig. 1D), which is consistent with the TCGA database (Fig. S1B). The expression of CD8 was decreased in glioma grade IV tissues compared with control tissues (Fig. 1C). The positive correlation was found between AP-2α and CD8 expression in glioma grade III/IV tissues (Fig. 1E). Moreover, AP-2α^low^PD-L1^high^ was found in wild-type IDH1 gliomas and glioma subtypes (classical, mesenchymal and neural) (Fig. S1C, D). Next, AP-2α expression was decreased while PD-L1 expression was upregulated in glioma tissues by qRT-PCR analysis (Fig. 1F). In addition, AP-2α^low^PD-L1^high^ expression was detected in glioblastoma and glioma cell lines U87 and U251 (Fig. 1G, H). Thus, PD-L1 expression is negatively associated with AP-2α expression in gliomas.

### AP-2α binds to the *CD274* promoter and inhibits *PD-L1* transcription

**Fig. 2:**
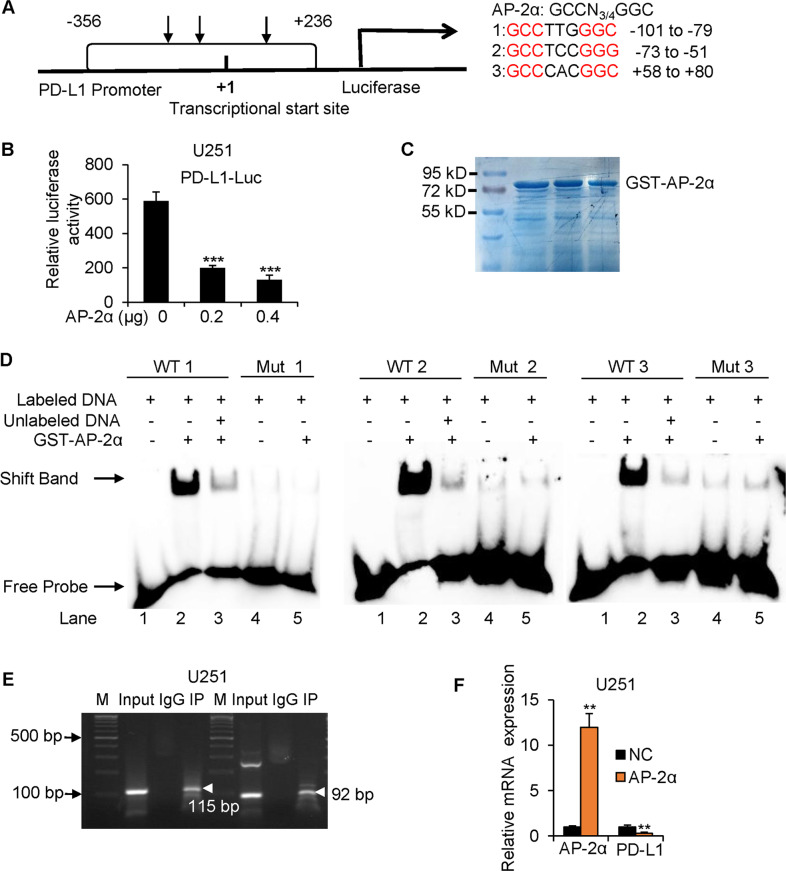
AP-2α binds directly to the promoter of the *CD274* gene. **A** The potential AP-2 binding sites in the promoter of the *CD274* gene. **B** The effects of AP-2α expression on the transcriptional activity of the *PD-L1* promoter in U251 cells. **C** Purified AP-2α proteins on SDS-PAGE gels. **D**, **E** EMSA and ChIP analysis of binding between AP-2α proteins and the *PD-L1* promoter in vitro and in U251 cells. **F** qRT-PCR analysis of the influences of AP-2α on *PD-L1* mRNA expression in U251 cells.

To investigate molecular mechanisms underlying AP-2α and PD-L1, we found three consensus AP-2-binding sites in the *PD-L1* promoter by JASPAR software (Fig. 2A). Luciferase assays revealed that AP-2α dose-dependently repressed reporter activities (Fig. 2B). A strong binding appeared between three labeled 23-bp probes containing AP-2-binding site and purified AP-2α proteins by EMSA (Fig. 2C, D). Competition binding assays revealed that the AP-2α/DNA complexes were reduced by the excess of the unlabeled probes. In contrast, mutant probes failed to bind with AP-2α proteins. Moreover, chromatin immunoprecipitation showed that *PD-L1* promoter with AP-2 sites could be immunoprecipitated in U251 cells (Fig. 2E). AP-2α decreased *PD-L1* mRNA levels in U251 cells (Fig. 2F). These data suggest that AP-2α binds to the *PD-L1* promoter and represses its transcription.

### AP-2α enhances the lysosome-dependent degradation of PD-L1

**Fig. 3:**
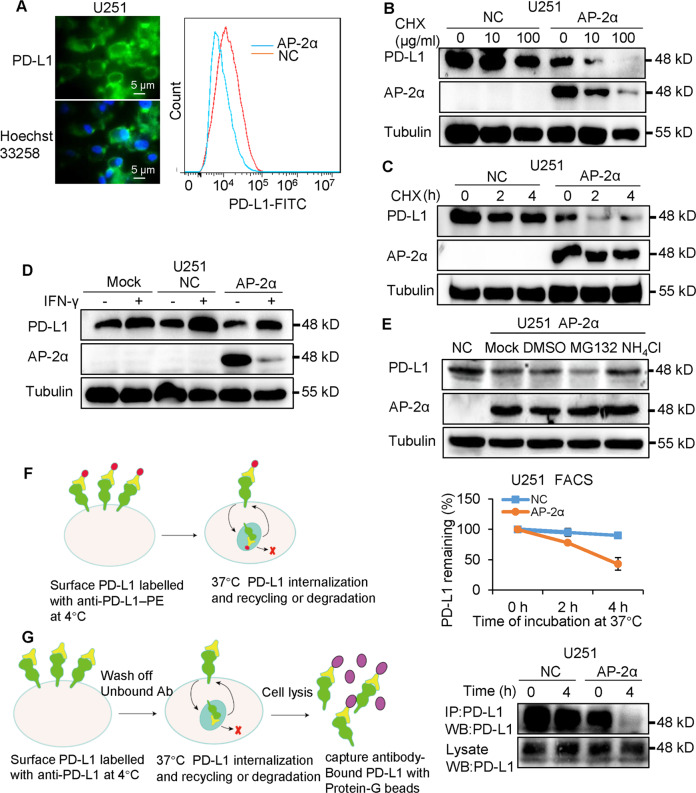
AP-2α enhances PD-L1 degradation in a lysosome-dependent manner in glioma cells. **A** Immunofluorescence analysis of PD-L1 protein localization and FACS analysis of the influences of AP-2α on PD-L1 abundance in U251 cells. Nuclei were stained with Hoechst 33258. **B**, **C** The effects of AP-2α on PD-L1 degradation in U251 cells upon treatment with CHX at different time points. **D** Western blotting of the effects of AP-2α proteins on constitutive/induced PD-L1 expression. **E** The PD-L1 degradation pathway is mediated by AP-2α. **F**, **G**, FACS analysis, and Co-IP assays of the effects of AP-2α on PD-L1 internalization and remaining in U251 cells.

PD-L1 was localized in the plasma membrane, and AP-2α decreased the abundance of PD-L1 proteins (Fig. 3A). A decrease in PD-L1 proteins was induced in AP-2α-overexpressing U251 cells treated with cycloheximide (CHX) at indicated timepoints than in control cells (Fig. 3B, C). Since IFN-γ induces PD-L1 expression [22], AP-2α could downregulate induced PD-L1 expression (Fig. 3D). AP-2α promoted the degradation of PD-L1 proteins, which was alleviated by the lysosome inhibitor NH_4_Cl not by the proteasome inhibitor MG132 (Fig. 3E). Similar results that AP-2α enhanced PD-L1 degradation were observed in U87 cells (Fig. S2A, B). We further investigated whether AP-2α mediated cell surface PD-L1 expression [23]. Surface PD-L1 degradation was increased while surface PD-L1 remained decreased in AP-2α-overexpressing cells by FACS analysis (Fig. 3F), suggesting that surface PD-L1 is increasingly internalized and degraded. And immunoprecipitation assays revealed that AP-2α decreases the amount of surface proteins PD-L1 endocytosed into U251 cells for 4 h (Fig. 3G). These studies suggested that AP-2α mediates PD-L1 stability in gliomas.

We then wondered whether the influence of AP-2α on PD-L1 expression is unique to glioblastoma, we evaluated AP-2α-regulated PD-L1 expression in hepatocellular cancer, breast cancer, and cervical cancer. AP-2α enhanced the degradation of PD-L1 proteins in MHCC97H cells, which was alleviated by the lysosome inhibitor NH_4_Cl (Fig. S2C–E). Consistently, a negative regulation between AP-2α and PD-L1 proteins exists in MDA-MB-231 cells and HeLa cells (Fig. S2F). These results indicated that AP-2α plays a ubiquitous role in mediating PD-L1 expression in certain malignant cancers.

### AP-2α enhances the ability of human CD8^+^ T cells to kill glioma cells in vitro

**Fig. 4:**
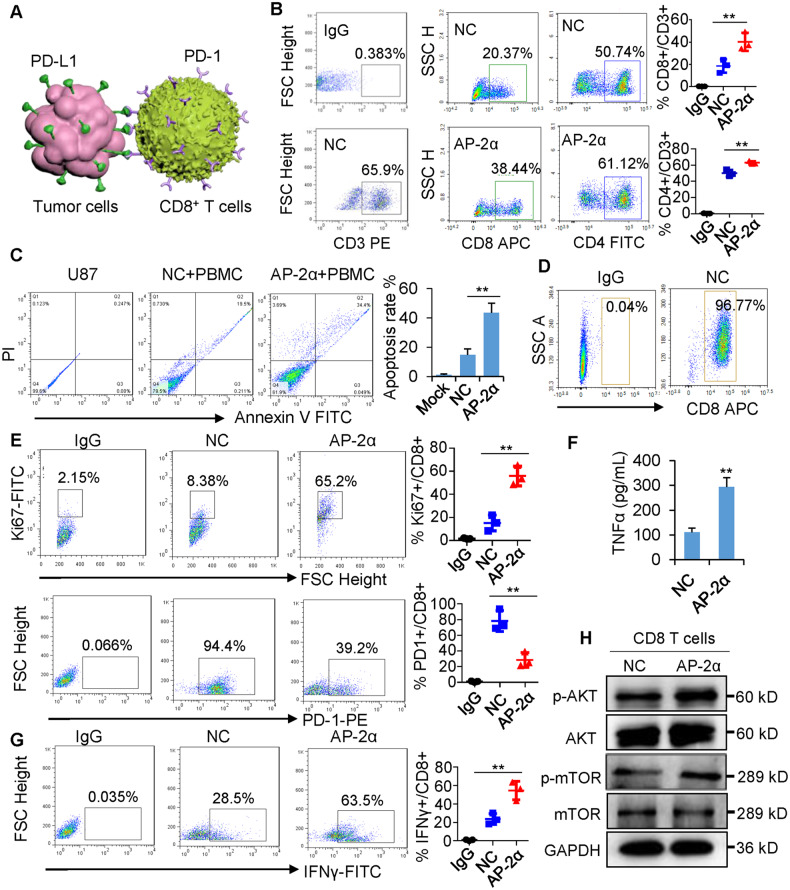
AP-2α in glioma cells enhances the killing activity of CD8^+^ T cells in the co-culture system. **A** CD8^+^ T lymphocyte-regulated cytotoxicity in PD-L1^+^ glioma cells. **B** CD3^+^, CD4^+^, and CD8^+^ proportions in PBMCs cocultured with U87 cells detected by flow cytometry. **C** U87 cells are killed by CD8^+^ T cells. **D** Purity of isolated CD8^+^ T cells from PBMCs by FACS analysis. **E** The expression of Ki67 and PD-1 in cocultured CD8^+^ T lymphocytes by FACS analysis. **F** ELISA analysis of cytokine TNF-α secreted into supernatant in the co-culture system, **G** IFN-γ expression in cocultured CD8^+^ T cells by FACS analysis. **H** Western blots of metabolic marker expression in cocultured CD8^+^ T cells.

Immune cell profiling revealed that AP-2α is correlated with the low score of M2 macrophages and the high score of CD8^+^ T cells in gliomas (Fig. S3A). AP-2α expression was positively related to the expression of cytotoxic T lymphocyte (CTL) markers in gliomas (Fig. S3B). PD-L1 on tumor cells binds with PD-1 on CD8^+^ T cells to enhance tumor immune evasion (Fig. 4A). We performed co-culture experiments to examine the effects of AP-2α in glioma cells on CD8^+^ T cell responses, PBMCs were co-cultured with U87 cells. The proportion of CD3^+^CD4^+^ and CD3^+^CD8^+^ subpopulations was increased when cocultured with AP-2α-overexpressing U87 cells (Fig. 4B). U87 cell apoptosis was improved compared with controls (Fig. 4C). To ensure the specific role of CD8^+^ T cells, we isolated CD8^+^ T cells with a percentage of 96.8% (Fig. 4D). FACS analysis showed that the abundance of Ki67 is enhanced while that of surface PD-1 is decreased in CD8^+^ T cells cocultured with AP-2α-overexpressing U87 cells (Fig. 4E), indicating proliferation of CD8^+^ T cells. In addition, the co-culture of CD8^+^ T cells with AP-2α-overexpressing U87 cells showed increased levels of TNFα and IFNγ to promote T lymphocyte activation (Fig. 4F, G) and activated AKT/mTOR pathway to enhance the metabolic program in potentiated CD8^+^ T cells (Fig. 4H). Therefore, AP-2α in gliomas enhanced the cytotoxicity of CD8^+^ T cells.

### AP-2α suppresses tumor progression and promotes anti-tumor immune response of anti-PD-1 antibodies

**Fig. 5:**
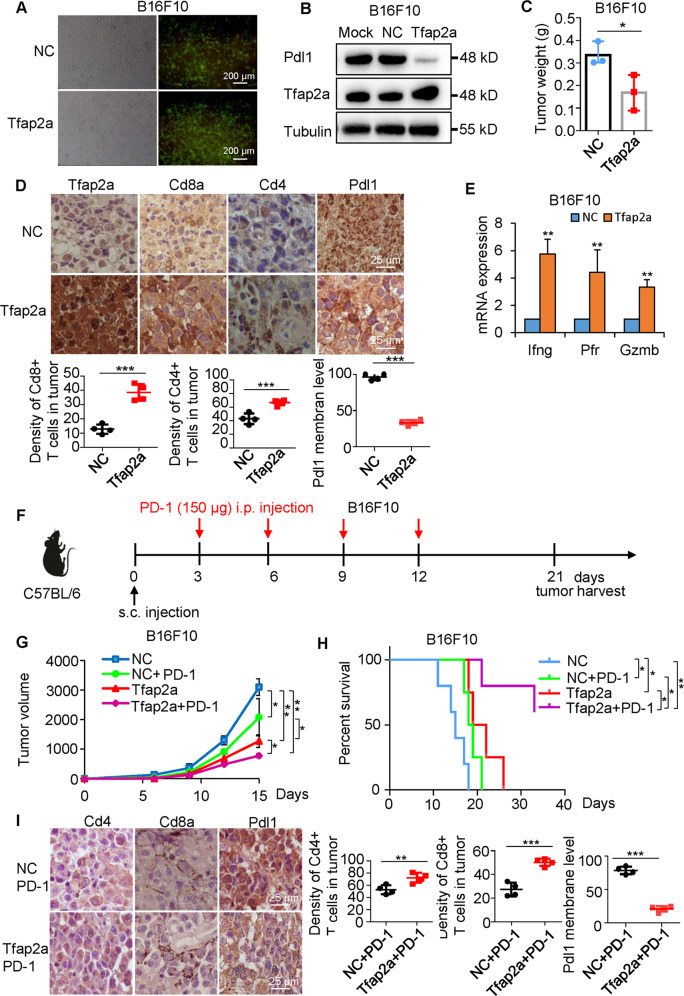
Tfap2a promotes anti-tumor immune response in B16F10 tumor-bearing mice. **A** Fluorescence images showing Tfap2a overexpression in B16F10 cells. **B** The effects of Tfap2a on Pdl1 proteins in B16F10 cells. **C** The effects of Tfap2a on the weights of subcutaneous B16F10 tumors. **D** The effects of Tfap2a on the expression of Pdl1 and Cd8a in B16F10 tumors by IHC analysis. **E** qRT-PCR analysis of Tfap2a and CTL markers in B16F10 tumors. **F** Combined therapeutics of Tfap2a overexpression and anti-PD-1 antibodies in B16F10 tumor-bearing mice. **G**, **H**, The effects of Tfap2a overexpression, anti-PD-1 antibodies or both on tumor volume and the survival of B16F10-bearing mice. **I** The effects of anti-PD-1 antibodies or Tfap2a/PD-1 abs on Cd8a and Pdl1 expression in B16F10 tumors.

We next sought to demonstrate that AP-2α-inhibited PD-L1 expression promotes antitumor immunity in vivo. Tfap2a-overexpressing stable B16F10 cells were inoculated subcutaneously into randomized BALB/c mice (Fig. 5A, B), Tfap2a suppressed tumor size (Fig. 5C and Fig. S4A). IHC staining revealed that Tfap2a decreases Pdl1 expression but improves the density of Cd4^+^ and Cd8^+^ T cells in B16F10 tumor cells (Fig. 5D). The expression of Ifng, perforin (Pfr) and Gzmb was increased in Tfap2a-overexpressing B16F10 tissues (Fig. 5E), indicating the stimulation and antitumor immunity of tumor-infiltrating CD8^+^ T cells. To investigate the therapeutic significance of AP-2α with PD-1 antibodies (Fig. 5F), we found that the combined treatment results in less tumor volume and slows tumor development compared with single treatment or control (Fig. 5G and Fig. S4B). There was no difference in the body weights of all mice (Fig. S4C). Approximately 50% of mice survived 33 days with the combined treatment, but all mice injected with anti-PD-1 antibodies died within 21 days (Fig. 5H), indicating synergistic anti-tumor immunity. The density of Cd4^+^ and Cd8^+^ T cells in tumor cells and spleens was improved by combined treatment compared with anti-PD-1 antibodies (Fig. 5I and Fig. S4D).

Similar results were obtained in CT26 mouse models, Tfap2a slowed tumor progression (Fig. S5A–D) and decreased Pdl1 expression accompanied by increased Cd8^+^ T cells and effector molecules in CT26 tumor tissues (Fig. S5E, F). In CT26 mouse models, the combination treatment resulted in tumor regression and prolonged the mouse survival compared with single treatment or control (Fig. S6A–D) and improved Cd8^+^ T cell function in tumor cells and spleens compared with anti-PD-1 antibodies (Fig. S6F, G) but all mouse weights remained unchanged (Fig. S6E). Therefore, AP-2α, in combination with immune checkpoint blockade, may have the best efficacy.

We next wondered whether AP-2α-mediated tumor immunity depends on CD8^+^ T cells, 6-week-old C57BL/6 mice were depleted of CD8^+^ T cells by anti-CD8 monoclonal antibody (Fig. S7A). Splenic Cd8^+^ T cells were stained in B16F10 mouse models, confirming Cd8 exhaustion (Fig. S7B). The Tfap2a-induced tumor regression was attenuated by Cd8 neutralization (Fig. S7C–E). In conclusion, AP-2α-mediated tumor shrinkage is dependent on CTLs.

### EZH2/H3K27me3/DNMT1 complex enhances *AP-2α* methylation in gliomas

**Fig. 6:**
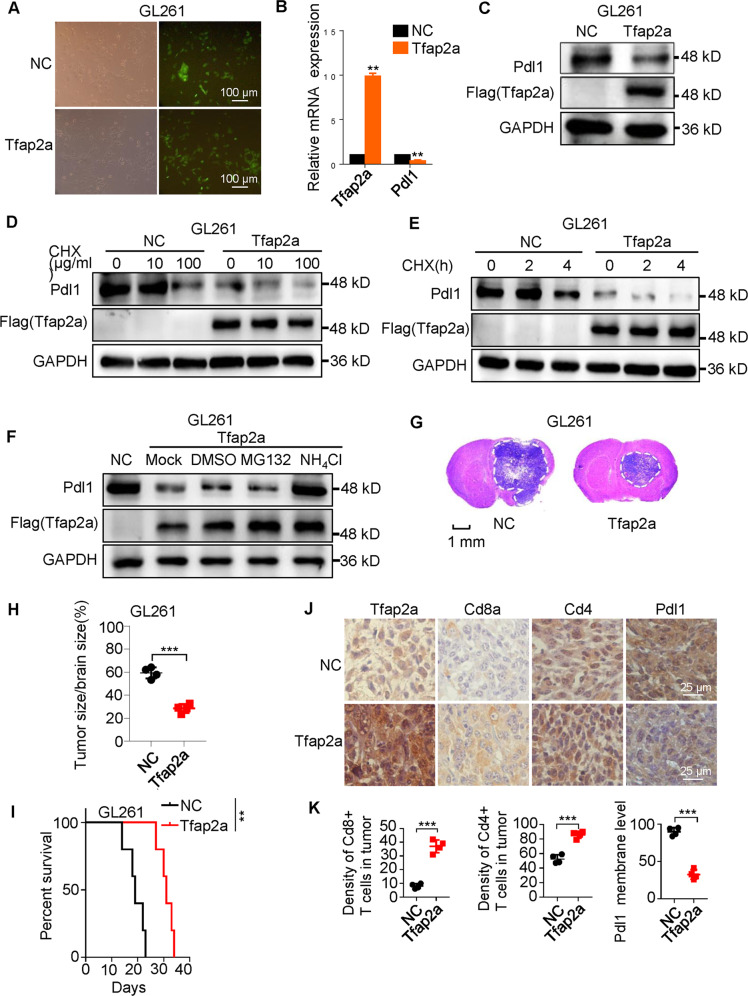
Tfap2a promotes anti-tumor immune response in GL261 tumor models. **A** Fluorescence images of Tfap2a-overexpressing GL261 cells. **B** The effects of Tfap2a on *Pdl1* mRNA expression in GL261 cells. **C** The effects of Tfap2a on Pdl1 protein levels in GL261 cells. **D**, **E** The effects of Tfap2a on Pdl1 expression in GL261 cells treated with different concentrations of CHX at different time points. **F** The effect of Tfap2a on the Pdl1 degradation pathway in GL261 cells. **G**–**I** The effects of Tfap2a on the intracranial GL261 tumors and mouse survival. **J** IHC analysis of the effects of Tfap2a on Pdl1, Cd8, and Cd4 expression in GL261 tumors.

Since AP-2α expression is lost in glioblastoma [24], we next address the molecular mechanisms of AP-2α expression. We first generated Tfap2a-overexpressing GL261 cell lines (Fig. 6A), confirmed that Tfap2a suppresses the transcription of the *Cd274* gene (Fig. 6B), enhances Pdl1 degradation (Fig. 6C–F). Tfap2a suppressed intracranial tumor growth (Fig. 6G, H), prolonged mouse survival (Fig. 6I), and improved CD4^+^ and CD8^+^ T abundance (Fig. 6J, K).

**Fig. 7:**
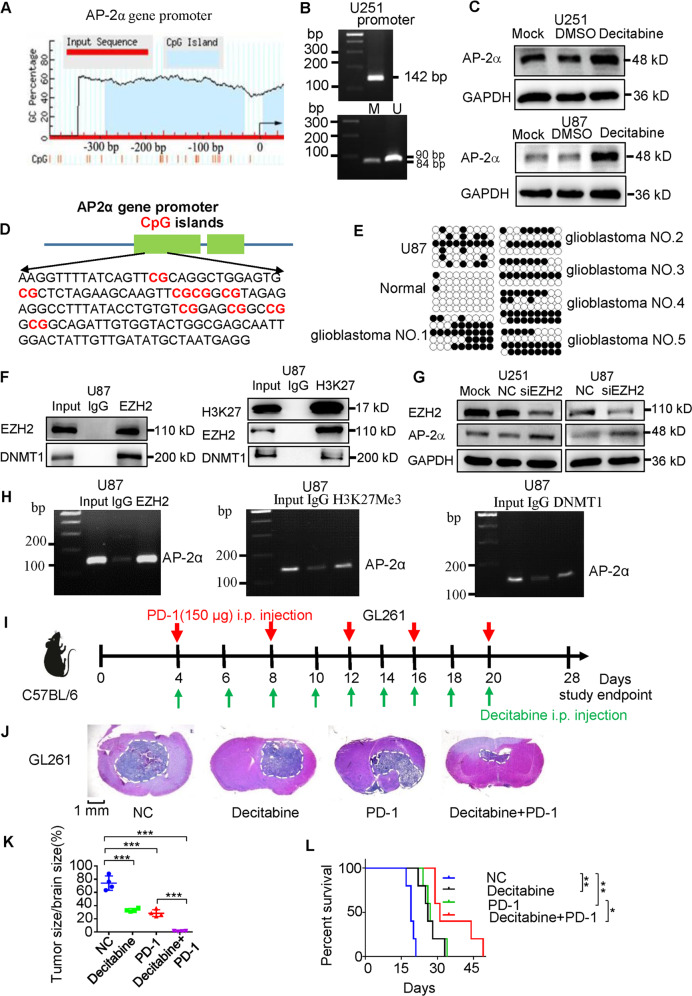
*AP-2α* demethylation and anti-PD1 antibodies promote an anti-glioma immune response in vivo. **A** The predicted methylated sites in the *AP-2α* promoter. **B** Methylation-specific PCR (MSP) of the *AP-2α* promoter treated with bisulfite. U unmethylated signal, M methylated signal. **C** The effects of Decitabine treatment on AP-2α expression in glioma cells. **D** CpG sites in the *AP-2α* promoter. **E** Methylated *AP-2α* sites were sequenced in glioma cell lines and glioblastoma tissues. • methylated sites, ° unmethylated sites. **F** Co-IP analysis of the interaction among EZH2, DNMT1, and H3K27me3 in U87 cells. **G** The influences of EZH2 knockdown on AP-2α expression in glioma cell lines. **H** ChIP analysis of the enrichment of EZH2, DNMT1, and H3K27me3 in the *AP-2α* promoter in U87 cells. **I** The combined strategies against intracranial GL261 tumors. **J**–**L** The influences of Decitabine and anti-PD-1 antibodies on intracranial tumor sizes and the survival of GL261-bearing mouse.

Epigenetic modifications are one of the major causes of gene silencing [25]. We found high CpG islands in the *AP-2α* promoter (Fig. 7A). Methylation-specific PCR (MSP) could amplify unmethylated and methylated PCR fragments, indicating *AP-2α* methylation in U251 cells (Fig. 7B). DNA methyltransferase (DNMT) inhibitor Decitabine improved AP-2α proteins (Fig. 7C). Bisulfite sequencing revealed CpG methylation of the *AP-2α* promoter in U87 cells and glioblastoma tissues (Fig. 7D, E). co-IP assays showed that Enhancer of Zeste Homolog 2 (EZH2), DNMT1 and H3K27me3 form an epigenetic modification complex (Fig. 7F). EZH2 Knockdown increased AP-2α expression in glioma cells (Fig. 7G). ChIP assays showed that the EZH2/H3K27me3/DNMT1 complex is enriched in the *AP-2α* promoter (Fig. 7H), suggesting that EZH2 recruits DNMT1 and affects H3K27 trimethylation in the *AP-2α* promoter. Therefore, these data indicated epigenetic silencing of the *AP-2α* gene.

Finally, we detected the effects of Decitabine on AP-2α expression in vivo. We treated GL261 tumor-bearing mice with Decitabine, anti-PD-1 antibodies, or both (Fig. 7I). Tumor volume treated with Decitabine or anti-PD-1 decreased by 47 and 53% compared with control tumors. Mouse tumors with combination therapy regressed by ~86% reduction (Fig. 7J-K). Moreover, approximately 40% of mice with combination treatment survived 43 days, whereas the medial survival of all other mice was 24.6 days (Fig. 7L). The combination treatment increased Tfap2a expression and improved Cd8^+^ T-cell abundance (Fig. S8A, B), indicating the combined efficacy against gliomas. Noteworthy, Decitabine upregulated Pdl1 expression even other gene expression in gliomas [26, 27], but Tfap2a overexpression could downregulate Decitabine-induced Pdl1 upregulation (Fig. S8C, D), elucidating the *AP-2α* methylation and AP-2α-suppressed PD-L1 expression in certain types of gliomas.

## Discussion

The importance of AP-2α in transcriptional regulation, carcinogenesis, and development has been highlighted [28–31], but the potential function of AP-2α in tumor immunity is still unknown. Here, we show that AP-2α expression was downregulated and PD-L1 expression was increased in glioblastoma. CD8^+^ T lymphocytes serve as cytotoxic effector cells against tumors [32], but CD8 expression remains at a low level in most gliomas [33]. Importantly, AP-2α proteins increased the percentage of effector CD8^+^ T cells in gliomas. IDH1 wildtype gliomas represent hypo-methylation and elevate the expression of the *PD-L1* gene [34], the TCGA dataset showed that AP-2α^low^/PD-L1^high^ was associated with wild-type IDH1 and glioma subtypes (classical, mesenchymal, neural), indicating the potential clinical significance of AP-2α in molecular classification and IDH1-wildtype glioma immunotherapy.

PD-L1 abundance was regulated at multiple levels, including transcription, post-transcription, and post-translation [35]. PD-L1 expression was regulated by transcription factors, including STAT3, NF-ҡB, HIF-1α, and miR-138-5p at the transcriptional and posttranscriptional levels [36–39]. Moreover, important proteins, including CMTM4/6, GSK3β, and CSN5, regulated PD-L1 stability via post-translational modifications [23, 40–42]. Although PTEN and FKBP51 regulated PD-L1 expression in gliomas [29, 43], the complicated regulatory mechanisms of PD-L1 expression need to be further investigated to accurately select patients and reduce resistance to PD-1 treatment in “cold” gliomas. Our results showed that AP-2α binds to the *PD-L1* promoter, inhibits its transcriptional activity, and enhances the lysosome-dependent degradation of PD-L1. In addition, AP-2α enhanced ubiquitous degradation of PD-L1 expression in several solid tumors [44], including hepatocellular carcinoma, cervical cancer, breast cancer, and mouse CT26 cells, B16F10 cells, which are commonly used in experimental tumor immunotherapy [45–47]. Therefore, AP-2α, which negatively regulates surface PD-L1 expression, may be considered a novel marker for PD-L1/PD-1-based immunotherapies.

Gliomas exhibit immune cell infiltrations with different functionality [48]. We confirmed that high expression of AP-2α is associated with increased frequency of CD8^+^ T cells in gliomas. Dysfunction of antitumor effector CD8^+^ T cells from the tumor microenvironment is a key feature of cancer [49]. Depletion of CD8^+^ T cells decreased cytotoxic function and produced few effector cytokines [50]. A co-culture system with CD8^+^ T cells and glioma cells decreased the proliferation of AP-2α-overexpressing U87 cells, but increased Ki67 expression and cytokines TNFα and IFNγ secretion, decreased PD-1 levels in CD8^+^ T cells in vitro. Tfap2a could decrease Pdl1 expression in tumor cells, increase the infiltration of Cd8^+^ T cells, enhance effector molecule release, and suppress tumor growth in vivo. Anti-CD8 antibody-mediated depletion showed that AP-2α-regulated PD-L1 expression in tumor immunogenicity depends on improved cytotoxic T cell activity. As expected [51], Tfap2a combined with anti-PD-1 antibodies [47] to improve Cd8^+^ T cell infiltration and inhibit tumor progression. Overall, the combination of AP-2α and PD-1 blockade could improve clinical efficacy in cancer patients.

The expression of AP-2α was lost in 99% of glioblastomas [24]. However, the critical mechanism of AP-2α downregulation in gliomas was unclear. Histone methyltransferase EZH2 could recruit DNMT to a target promoter and catalyze H3K27 trimethylation to enhance epigenetic silencing [52, 53]. We demonstrated the crosstalk between the EZH2/H3K27me3/DNMT1 complex and *AP-2α* methylation in gliomas (Fig. S9), suggesting that *AP-2α* methylation may be a critical epigenetic mechanism in glioblastoma. Depletion of Ezh2 was correlated with disruption of CD8^+^ Teff cell differentiation [54], suggesting synergistic regulation between epigenetic modification, antitumor immunity, and tumor signaling pathways. Decitabine treatment in mouse glioma models increased CTL-mediated killing [55]. EZH2 siRNA or Decitabine could increase AP-2α expression and boost anti-tumor immunity, which partly explains the low response of glioma immunotherapy. GL261 Tumor volume was decreased upon treatment with the anti-PD-1 antibodies, as reported [51], and combined therapy with Decitabine and immune checkpoint blockade promoted the greatest extent of regression. Although Decitabine as a non-specific demethylating agent, upregulated Pdl1 expression [56], Tfap2a could downregulate Decitabine-induced Pdl1 upregulation. However, the detailed regulation network needs to be deeply investigated to optimize the safety and efficacy of Decitabine.

Taken together, these studies describe AP-2α as a novel PD-L1 regulator in anti-glioma immunity and has ubiquitous therapeutic implications for solid tumors. Understanding the mechanism of AP-2α may provide more valuable information to control anti-tumor immunity and suggest new combined strategies to combat malignant tumors.

## Materials and methods

Details of the following “Materials and methods” were described in the Supplemental Materials and Methods.

### Immunohistochemical (IHC) analysis

The experiments were approved by the Ethics Committee of Hunan Normal University, and informed consent was obtained from all patients. Polyformalin-fixed paraffin-embedded tissues were performed.

### RNA extraction and qRT-PCR

Total RNA was extracted using TRIzol reagent and reverse transcribed into cDNA. SYBR green (Invitrogen)-based real-time PCR was carried out using ABI 7900 thermocycler.

### Cell culture and transfection

Tumor cell lines were cultured in Dulbecco’s modified Eagle’s medium with fetal bovine serum. PBMCs were cultured in RPMI 1640 medium.

### Plasmid construction

Plasmids were constructed and sequenced by the Sanger method.

### Generation of AP-2α-overexpressing cell lines

Lentiviral particles were generated, and tumor cells were infected and screened according to standard procedures.

### Immunoblotting, endogenous co-IP, luciferase assays, EMSA, chromatin immunoprecipitation

For immunoblotting, cells were lysed in RIPA buffer and detected.

coIP analysis was performed following the manufacturer’s protocol.

For the luciferase assays, the cells were cultured, and the expression of the luciferase reporter gene was measured.

The EMSA was carried out following standard procedures.

ChIP was performed using an EZ-ChIP assay kit.

### Immunofluorescence

Cells were treated and stained, fluorescence signals were analyzed using a fluorescence microscope.

### Flow cytometry

Cells were stained and detected on a FACSCalibur.

PBMCs were isolated by the Ficoll method and added to glioma cells. Cell apoptosis was analyzed by an Annexin V-FITC/PI assay. The proportions of stained T lymphocytes were measured by FACS analysis.

CD8^+^ T cells were fixed, blocked, and stained, followed by FACS analysis.

### In vivo functional assays

For mouse models, tumor cells were subcutaneously or intracranially injected into randomized mice. Anti-CD8 monoclonal antibodies, anti-PD-1 antibodies, or Decitabine were administrated by intraperitoneal injection into tumor models.

### Detection and sequencing of methylation sites in the *AP-2α* promoter

Genomic DNA was modified by bisulfite treatment, amplified, inserted into T-vector, and sequenced.

### Statistical analysis

Statistical analyzes were conducted using GraphPad software (San Diego, California, USA). *P* values of <0.05 were considered significant.

## Supplementary information

checklist

Supplemental materials and methods

Original Data File

## Data Availability

All data generated or analyzed during this study are available from the corresponding author upon reasonable request.
