# Supplementary material for: Epigenetically modified AP-2α by DNA methyltransferase facilitates glioma immune evasion by upregulating PD-L1 expression
Source: Cell Death Dis. 2023 Jun 17;14(6):365. doi: 10.1038/s41419-023-05878-x (PMC10276877; doi:10.1038/s41419-023-05878-x)
Supplement: Supplementary file 2 — Supplemental materials and methods [file 41419_2023_5878_MOESM2_ESM.docx]

**SUPPLEMENTAL MATERIALS AND METHODS**

**Immunohistochemical (IHC) analysis**

Brain tissues were examined for IHC analysis. The experiments were approved by the Ethics Committee of Hunan Normal University, and informed consent was obtained from all patients. Polyformalin-fixed paraffin-embedded (FFPE) tissues were performed as described [S[1](#_ENREF_1), [2](#_ENREF_2)]. Primary antibodies are AP-2α (3B5, Santa Cruz Biotech, Santa Cruz, CA) (1:200), PD-L1 (ab228415, Abcam, Cambridge, UK) (1:200), CD8α (70306, Cell Signaling, Danvers, MA) (1:200), or IgG control (ab37355, Abcam) (1:500).

**RNA extraction and qRT-PCR**

Total RNA was extracted from glioma tissues and cell lines using TRIzol reagent (Thermo Fisher Scientific), and reverse transcribed into cDNA using MMLV RTase and a random primer (Sangon). SYBR green (Invitrogen)-based realtime PCR was carried out using ABI 7900 thermocycler (Thermo Fisher Scientific) [S[1](#_ENREF_1)]. Reactions were incubated in a 384-well plate at 95 °C for 5 min, followed by 35 cycles of 95 °C for 20 s and 60 °C for 30 s. The PCR primers were listed in Table S1. Relative expression levels of genes were indicated by 2^−ΔΔCt^ compared with controls.

**Cell culture and transfection**

Human glioma cell lines A172, U251, SHG44, U87, G261, B16F10, CT26, MHCC97H, MDA-MB-231, HeLa, HEK293 and 293T cells from the American Type Culture Collection (ATCC) were purchased and authenticated, and cultured in Dulbecco's modified Eagle's medium (DMEM, Thermo Scientific, Waltham, MA, USA) with fetal bovine serum (FBS, Thermo Scientific), penicillin G and streptomycin (Invitrogen Life Technologies, Carlsbad, CA, USA). All cells were kept in a humidified atmosphere with 5% CO_2_ at 37˚C as described [S[3](#_ENREF_3)]. PBMCs from healthy donors were isolated by the Ficoll method and stimulated with 5 µM PHA and 75 IU/mL IL-2 in complete RPMI 1640 medium for 3 days. Plasmids or siRNAs were transfected into cells using Lipofectamine 3000 (Invitrogen) [S[1](#_ENREF_1)].

**Plasmid construction**

The homo sapiens *AP-2α* full-length cDNA and mus *Tfap2a* cDNA were subcloned into the lentiviral vector pEZ-Lv105-Puromycin (GeneCopoeia), pEZ-Lv105-GFP-Puromycin served as a negative control (NC) [S[3](#_ENREF_3)]. The promoter region of the *PD-L1* gene was amplified from HEK293 cells and cloned into the pGL3-basic vector (Promega Corporation, Madison, WI, USA). All constructs were sequenced by the Sanger method (Sangon Biotech, Co., Ltd) for verification.

**Generation of AP-2α or Tfap2a-overexpressing cell lines**

Lentiviral particles were generated as described [S[2](#_ENREF_2)]. Tumor cell lines were infected with AP-2α/Tfap2a-lentivirus and NC-lentivirus and examined by the fluorescence microscope after screening stable tumor cell lines with puromycin.

**Immunoblotting, endogenous co-IP, luciferase assays, EMSA, chromatin immunoprecipitation**

For Immunoblotting, harvested or treated cultured cells and glioma tissues were lysed in RIPA buffer [S[3](#_ENREF_3)]. Information of antibodies used were followed, mouse monoclonal antibodies against AP-2α (3B5), PD-L1 (28076-1-AP, proteintech), Tubulin (AF7010, Affinity Biosciences) and GAPDH (AP0063, Abbkine) were used. Rabbit polyclonal antibodies against EZH2 (A16846), DNMT1 (A16729), H3K27Me3 (A2363), mTOR (A2445) and p-mTOR (AP0094) were from ABclonal Technology (MA, USA). Rabbit antibodies against AKT (D260001, BBI-Life-Sciences) and p-AKT (A11027, ABclonal) were used. HRP-conjugated goat anti-mouse and goat anti-rabbit secondary antibodies were from ABclonal. Signal was detected using the SuperSignal West Pico chemiluminescent substrate (Thermo Fisher Scientific, Rockford, IL, USA) and visualized using the tanon system (Bio-Tanon, Shanghai, China).

coIP analysis was performed as follows, glioma cells were grown, collected and lysed in RIPA buffer with protease inhibitors [S[4](#_ENREF_4)]. Lysates were precleared and then immunoprecipitated with rabbit polyclonal antibodies against EZH2 (A16846) or H3K27Me3 (A2363) and protein A/G PLUS agarose (Santa Cruz Biotech). After washing, the precipitates were detected by Western blotting using rabbit monoclonal antibodies against EZH2 (D2C9), DNMT1 (D63A6) and H3K27Me3 (C36B11, Cell signaling Technology). To immunoprecipitate cell surface PD-L1, live U251 cells were incubated with the antibody against PD-L1 (2 μg/mL, 28076-1-AP) for 1 h on ice. After washing three times, cells were resuspended in prewarmed complete DMEM medium and incubated for the indicated time at 37 °C. Cells were put back on ice, washed and lysed. Lysates were incubated with protein G dynabeads for 2 h at 4 °C to capture antibody-bound PD-L1. After five washes, samples were eluted in SDS sample buffer, denatured, and detected by immunoblotting.

For the luciferase assays, the cells were cultured for 36 h after transfection and the expression of the luciferase reporter gene was measured using the luciferase reporter assay system (Promega).

EMSA experiments were carried out as follows. The GST-AP-2α fusion protein was purified [S[5](#_ENREF_5)]. The probes including AP-2 binding sites in the *PD-L1* promoter region were labeled with biotin. The sequences of the probes are shown in Table S1. According to the manufacturer's instructions of the EMSA Kit (Beyotime, Shanghai, China) [S[3](#_ENREF_3)], the binding reaction was performed in a mixture containing 1 pmol of biotin-labelled wild-type or mutant sequences and 3 μg GST-AP-2α in 15 μl binding buffer for 25 min. The mixtures were run on a 4% non-denatured gel followed by the chromogenic reaction.

ChIP was performed using an EZ-ChIP assay kit from Beyotim (SH, CHN) following the manufacturer’s protocol [S[6](#_ENREF_6)]. 2×10^7^ cells were cross-linked with 1% formaldehyde, washed and lysed. Chromatin was sonicated into fragments with approximately 1000 base pairs. The extracts were precleaned and then immunoprecipitated with 2 μg of rabbit polyclonal antibodies against AP-2α, EZH2, H3K27Me3, DNMT1 or preimmune IgG (sc-66931, Santa Cruz Biotech) for overnight at 4°C. Forty μl Protein G Agarose was added for 2 h incubation. DNA was eluted from protein-bound beads, reverse-crosslinked and purified. PCR was carried out using ExTaq DNA polymerase (ABclonal) with 34 cycles. Primers used for PCR amplification were listed in Table S1.

**Immunofluorescence**

U251 cells were cultured in a 24-well plate and grown to 60% confluence. After 18 h, cells were treated as previously described [S[7](#_ENREF_7)]. Rabbit polyclonal anti-PD-L1 antibodies (A1645, Abclonal) were used as primary antibodies, while Alexa Fluor 488 phalloidin (Thermo Fisher Scientific) were used as secondary antibodies. The nucleus was stained using Hoechst 33258 (Beyotime). Fluorescence signals were analyzed using a fluorescence microscope (Zeiss Axioskop-2).

**Flow cytometry**

Glioma cells were stained for 20 min in PBS plus 1% FBS for PD-L1 [S[8](#_ENREF_8)]. Antibodies were IgG-FITC (11-4321-80, Invitrogen), and anti-PD-L1-FITC (12-5983-41, invitrogen). For internalization assay [S[7](#_ENREF_7)], U251 cells were labeled with anti-PD-L1-PE (12-5983-42, eBioscience) for 45 min on ice and then incubated at 37 °C for the indicated time detected on a FACSCalibur (BD Biosciences) and FACS data was read by FlowJo software.

To detect the tumor-killing effect of CD8^+^ T cells, PBMCs were isolated by the Ficoll method and added to glioma cells to maintain an effector-to-target (2:1) for 10 h. Glioma cell apoptosis was analyzed by an Annexin V-FITC/PI assay [S[3](#_ENREF_3)]. The proportions of CD3**^+^**, CD4**^+^** and CD8**^+^** T-lymphocytes were measured with anti-CD3-PE (PE-65133), anti-CD8-APC (APC-65144) and anti-CD4-FITC (FITC-65413) (proteintech) by FACS analysis [S[9](#_ENREF_9)].

For the phenotype of CD8^+^ T cells, CD8^+^ T cells were isolated using the Human CD8^+^ Magnetic Beads Kit (KMS003, proteintech) after coculture. CD8^+^ T cells were fixed, blocked and stained with anti-CD8-APC, anti-PD-1-PE (12-9969-41), anti-Ki67-FITC (11-5698-80) and anti-IFNγ-FITC (11-7139-82) (invitrogen) after permeabilization for 30 min followed by FACS analysis [S[8](#_ENREF_8)]. Supernatant TNF-α content was quantified using Human TNF-α ELISA MAX Deluxe (Biolegend) [S[8](#_ENREF_8)].

***In vivo* functional assays**

For the B16F10 and CT26 mouse models, 5×10^6^ tumor cells were injected subcutaneously into randomized 5-week-old female C57BL/6J mice and Balb/c mice. Antibody blockade experiments were performed intraperitoneally (IP) at 150 μg/mouse with anti-CD8 monoclonal antibody (YTS169.4, BioXCell) and control antibody (LTF-2, BioXCell) [S[10](#_ENREF_10)]. Anti-CD8 antibodies were administrated 1 day before B16F10 tumor implantation (day -1), day 0, and then every 3 days. Anti-PD-1 antibodies (150 µg/mouse, RPM1-14, BioXCell) were administrated by intraperitoneal injection on days 3/6/9/12 for B16F10 models and on days 10/13/16 for CT26 models [S[10](#_ENREF_10)]. For the combined therapy, C57BL/6J mice were intracranial injected with 5 ×10^5^ GL261 cells. Mice were treated with Decitabine (3.75 mg/kg) (A1906, Sigma, St. Louis, Mo, USA), monoclonal anti-PD-1 antibodies (150 µg/mouse), or both treatments. Tumor weight, volume and mouse survival were recorded, and tumor tissues were analyzed [S[3](#_ENREF_3)].

**Detection and sequencing of methylation sites in the *AP-2α* promoter**

CpG density of the *AP-2α* gene was performed using Online software (http://www.urogene.org/cgi-bin/methprimer/methprimer.cgi). Genomic DNA from glioma cells was modified by bisulfite treatment [S[11](#_ENREF_11)], amplified with primers (Table S1), inserted into T-vector, and sequenced. Glioma cells were treated with Decitabine for 48 h or transfected with EZH2 siRNAs detected by Western blotting.

**Statistical analysis**

The data indicating three independent experiments was expressed as mean ±SD. Statistical analyzes were conducted using GraphPad software (San Diego, California USA) and SPSS 20.0 (SPSS Inc., Chicago, Illinois USA). P values of < 0.05 were considered significant. The Student *t*-test and Fisher′s exact test compares gene expression between two datasets or more. The Kaplan-Meier method evaluates the survival.

**SUPPLEMENTAL FIGURE LEGENDS**

**
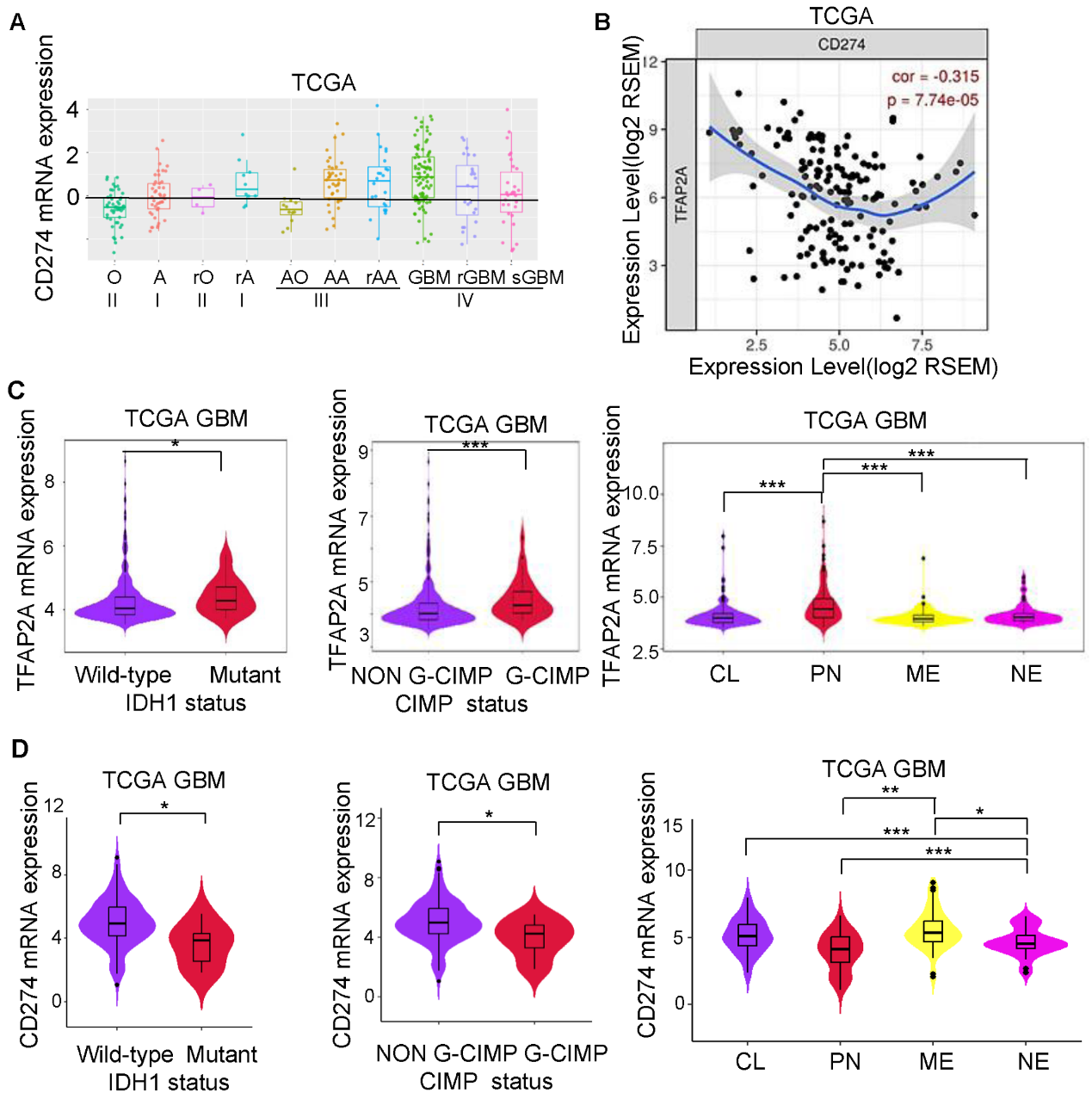
**

**Supplemental Figure 1 *AP-2α* expression and *CD274* expression in gliomas by bioinformatics analysis**

A, *CD274* expression in different grades of glioma tissues in the TCGA cohort. B, Correlation of *AP-2α* and *CD274* mRNA expression in the TCGA cohort. C-D, *AP-2α* and *CD274* expression in different types of glioma tissues.


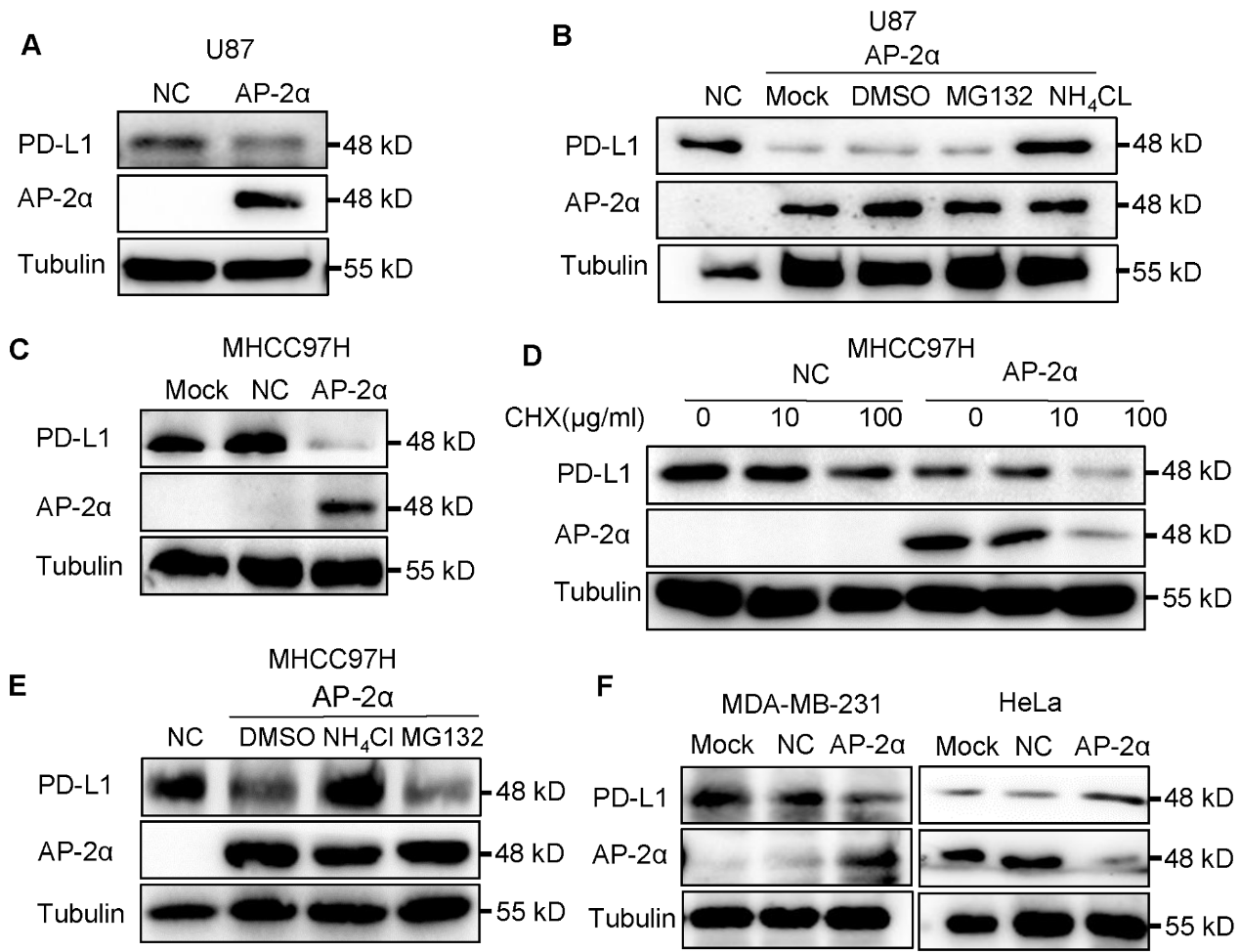


**Supplemental Figure 2 AP-2α enhances PD-L1 protein degradation in several cancer cell lines**

A, The effects of AP-2α on PD-L1 expression in U87 glioma cells. B, The effect of AP-2α on the PD-L1 degradation pathway in U87 cells. C, The effects of AP-2α on PD-L1 expression in MHCC97H cells. D, The effects of AP-2α on PD-L1 degradation in MHCC97H cells treated with different concentrations of CHX. E, The detection of AP-2α-mediated PD-L1 degradation pathway in MHCC97H cells. F, The effects of AP-2α overexpression and knockdown on PD-L1 expression in MDA-MB-231 and HeLa cells.


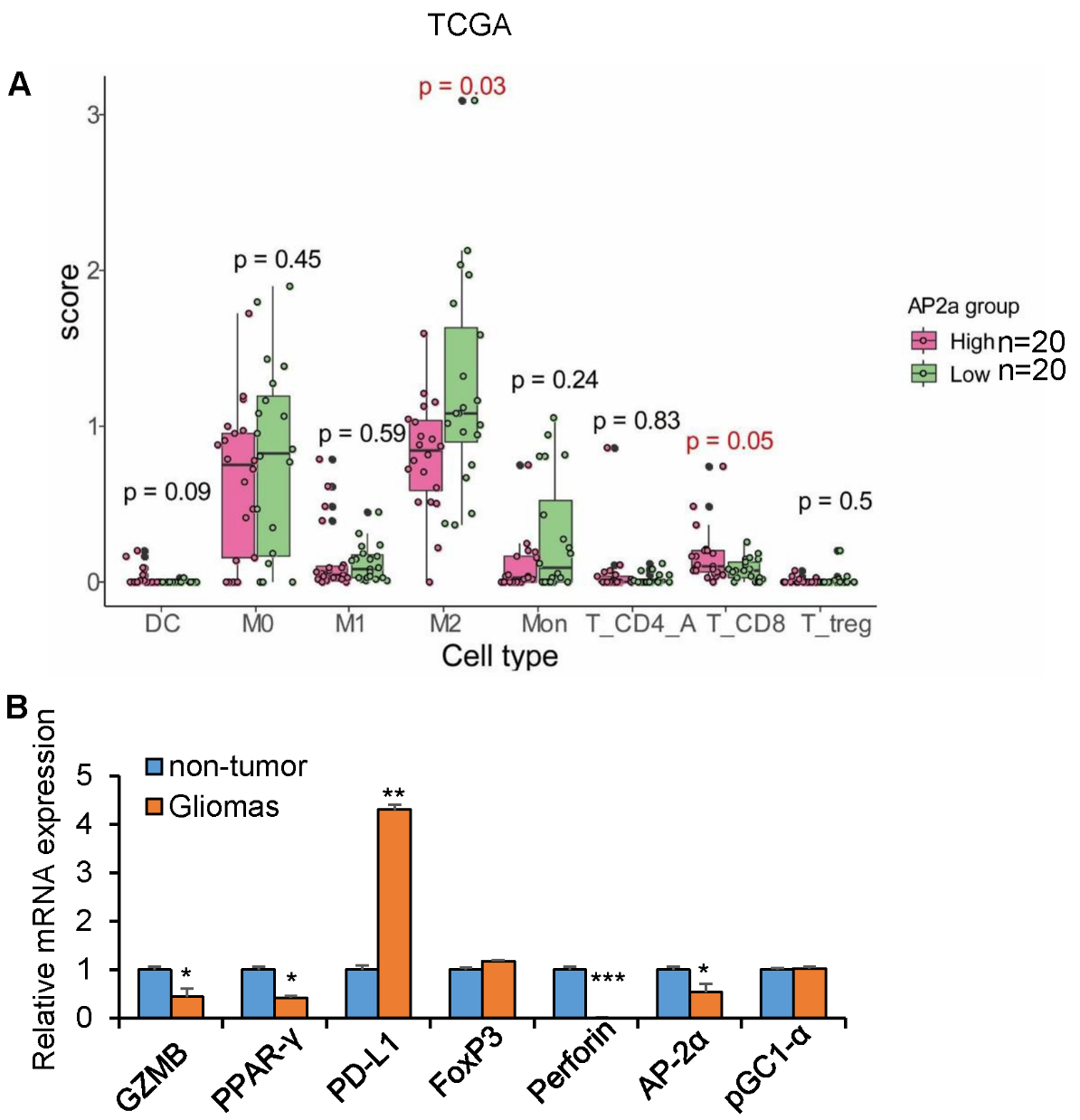


**Supplemental Figure 3 The expression *of AP-2α* and CTL markers in glioma tissues**

A, Correlation of *AP-2α* expression and immune cell infiltration analyzed by the CIBERSORTx method. B, qRT-PCR analysis of *AP-2α* and cytotoxic T lymphocyte markers in glioma grade IV tissues.

**
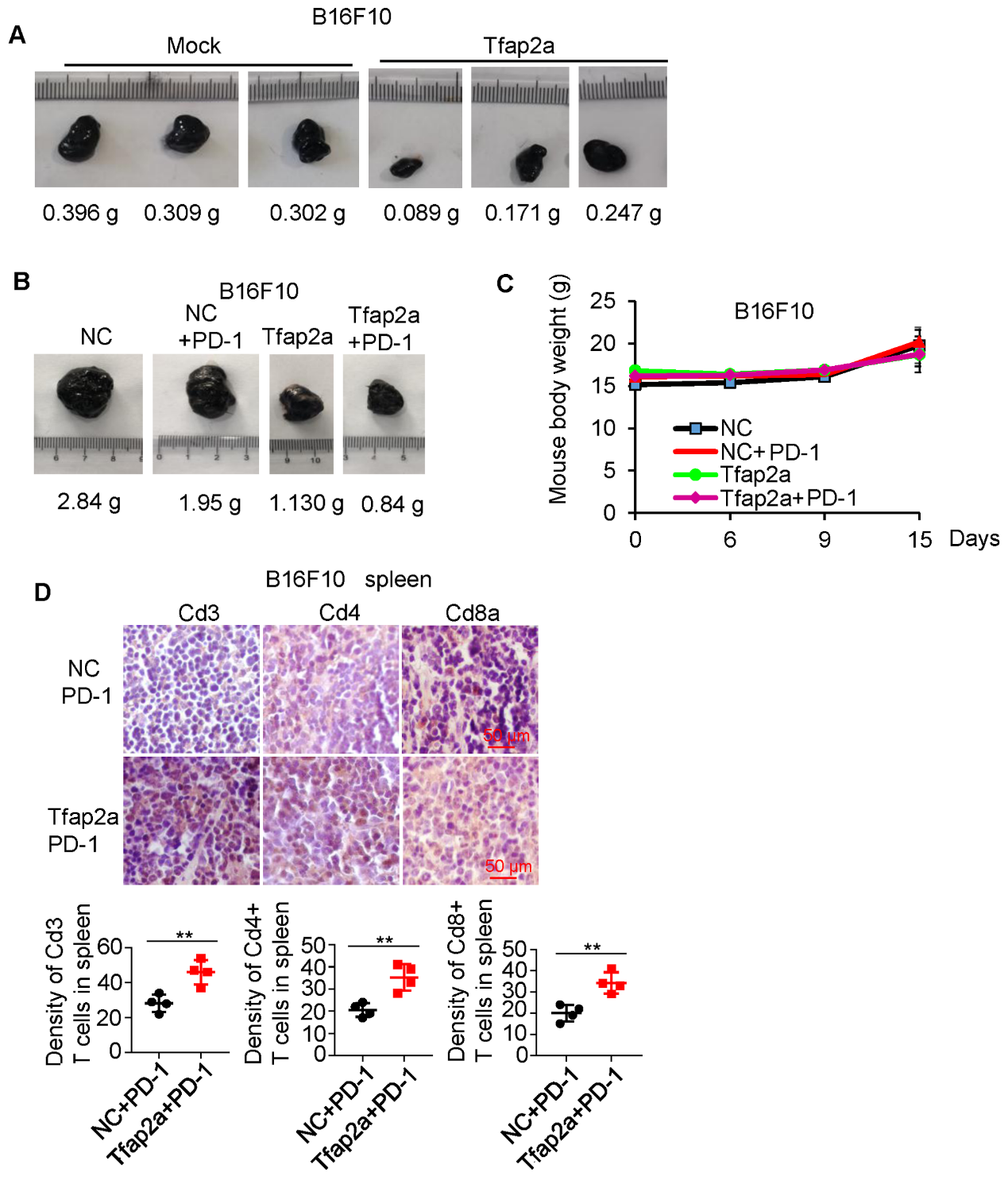
**

**Supplemental Figure 4 Tfap2a and anti-PD-1 antibodies promote anti-tumor immune response in B16F10 tumor model**

**A,** The representative figures showing the effects of Tfap2a on subcutaneous B16F10 tumors. B, The representative figures showing the effects of Tfap2a overexpression, anti-PD-1 antibodies or both on B16F10 tumor size. C, The effects of anti-PD-1 antibodies or Tfap2a/PD-1 abs on body weights of B16F10 tumor-bearing mouse. D, The effects of anti-PD-1 antibodies or Tfap2a/PD-1 abs on the expression of Cd3, Cd4 and Cd8a in the spleens of mice bearing subcutaneous B16F10 tumors.

**
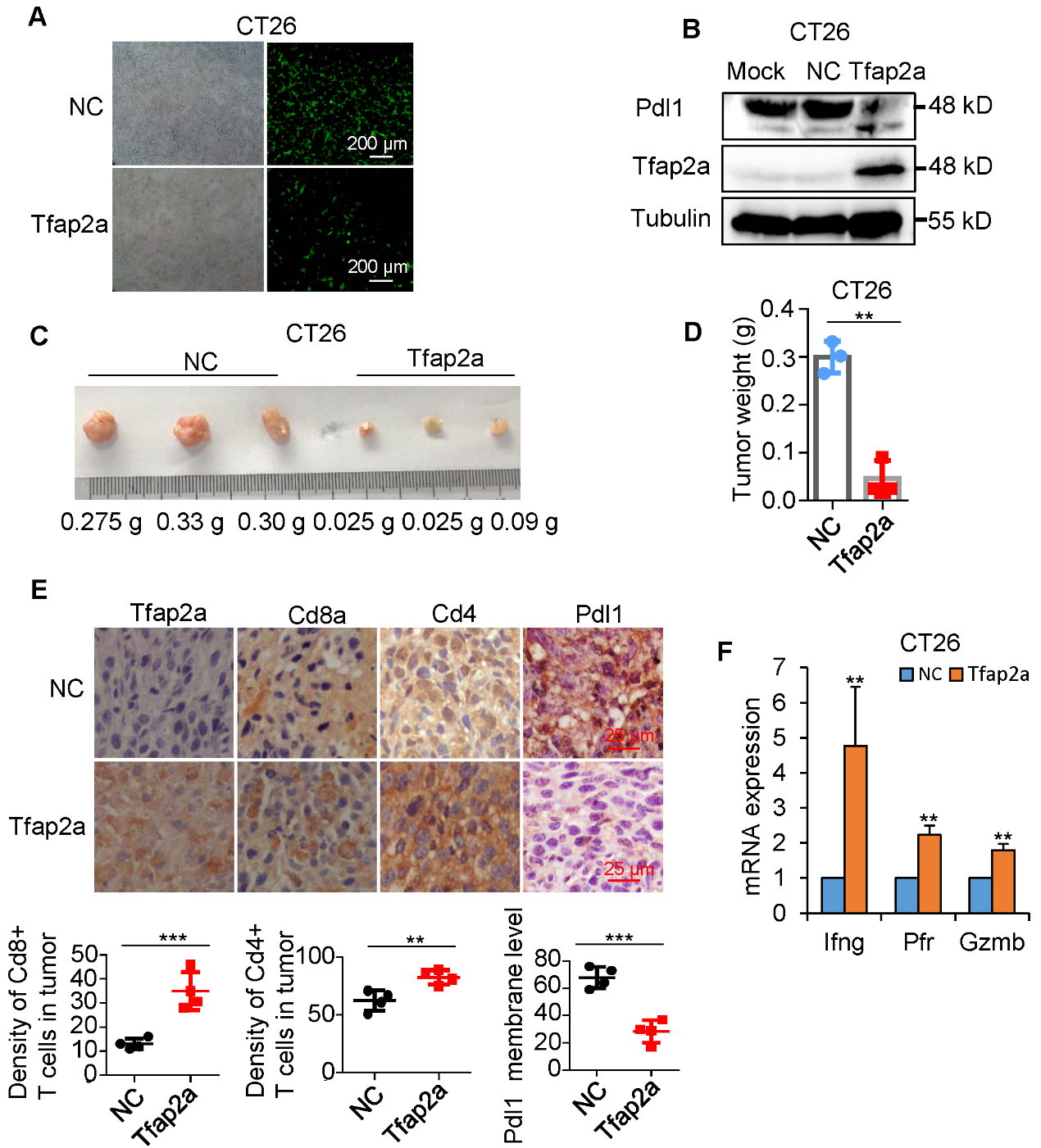
**

**Supplemental Figure 5 Tfap2a promotes anti-tumor immune response in CT26 tumor-bearing mouse**

A, Fluorescence images showing the overexpression of Tfap2a in CT26 cells. B, The effects of Tfap2a overexpression on Pdl1 expression in CT26 cells. C, The representative figures showing the effects of Tfap2a on subcutaneous CT26 tumors. D, The effects of Tfap2a on the weights of subcutaneous CT26 tumors, E, The effects of Tfap2a on the expression of Pdl1 and Cd8a in CT26 tumors by IHC analysis. F, qRT-PCR analysis of CTL markers in subcutaneous CT26 tumors.


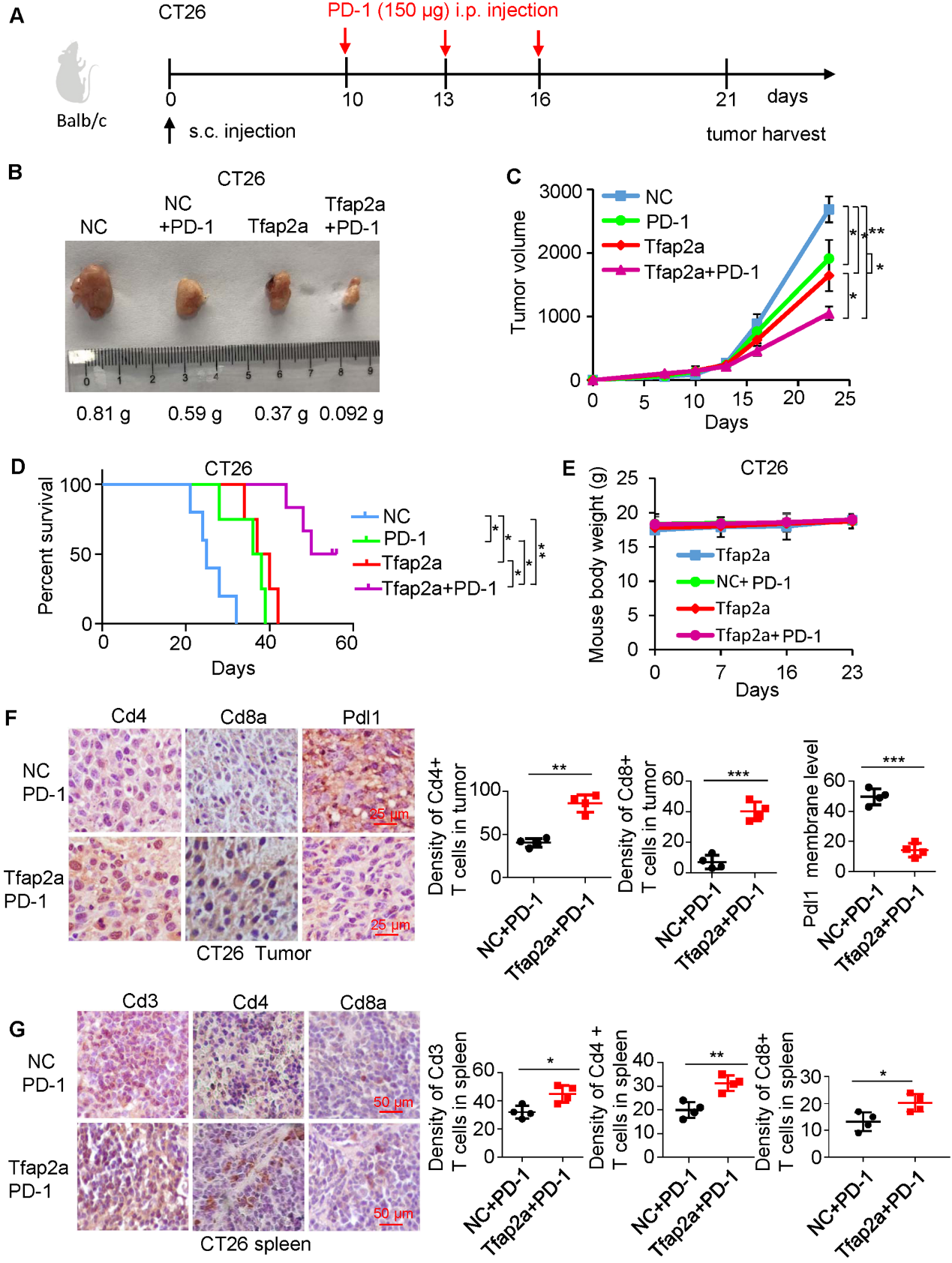


**Supplemental Figure 6 AP-2α and anti-PD-1 antibodies enhance the anti-tumor immune response in CT26 tumor models**

A, Combined therapeutic strategies showing Tfap2a overexpression and anti-PD-1 antibodies in mice bearing CT26 tumors. B, The representative images showing subcutaneous CT26 tumors under different treatments. C-E, The effects of Tfap2a overexpression, anti-PD-1 antibodies or both on CT26 tumor volume, the survival and body weights of BALB/c mice bearing CT26 tumors. F-G, The effects of anti-PD-1 antibodies or Tfap2a /PD-1 abs on the expression of Cd3, Cd4 and Cd8a in CT26 tumors and the spleens of subcutaneous CT26 tumor mice.

**
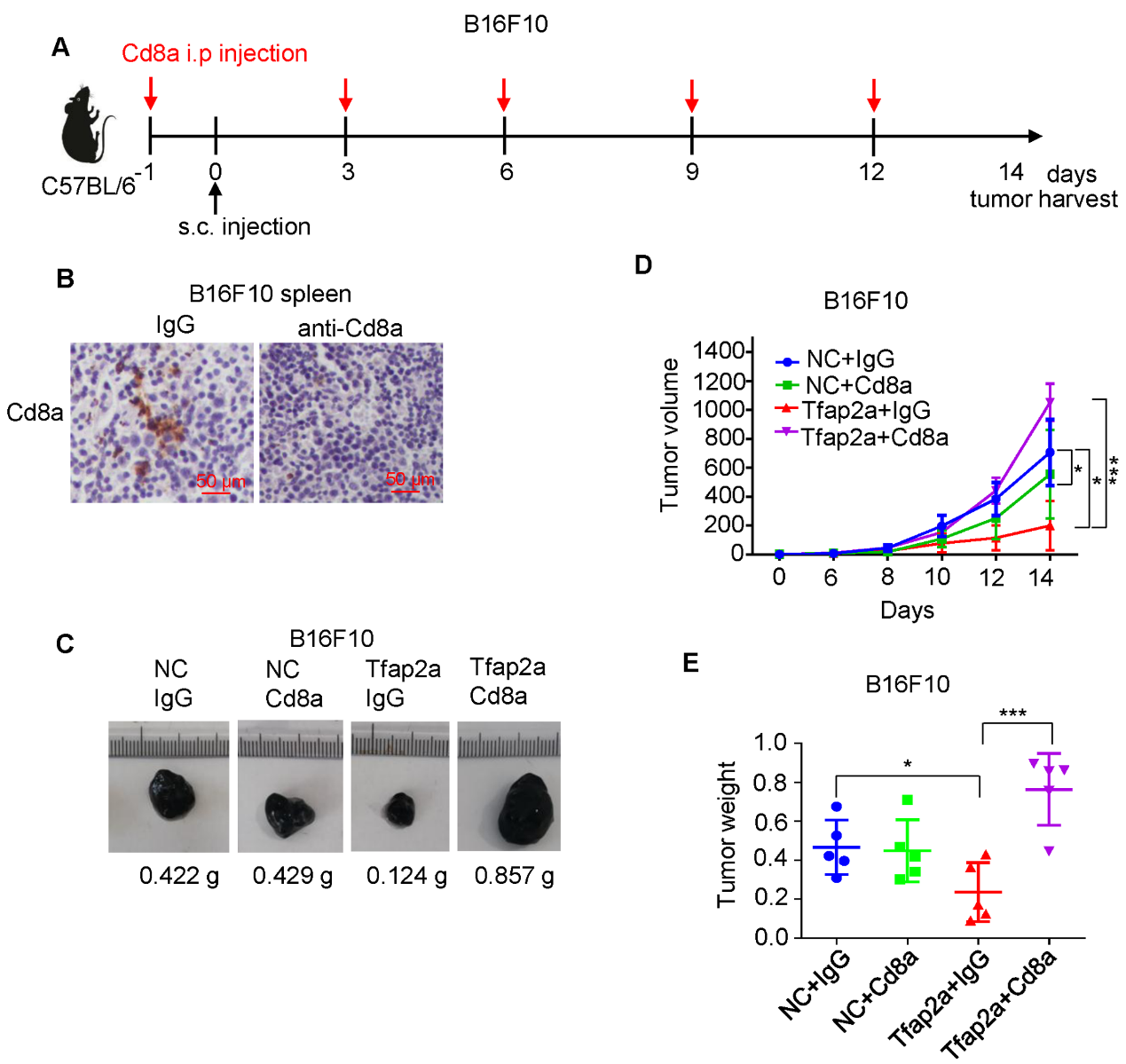
**

**Supplemental Figure 7 The anti-tumor immune activity of AP-2α is CD8^+^ T cell dependent**

A, The strategies of Cd8 depletion on subcutaneous B16F10 tumors. B, The percentage of Cd8^+^ T cells in the spleen confirmed by IHC analysis. C, The effects of Cd8 exhaustion on the size of mouse B16F10 tumors. D-E, The effects of Cd8 exhaustion on the volume and weights of subcutaneous B16F10 tumors inhibited by Tfap2a.


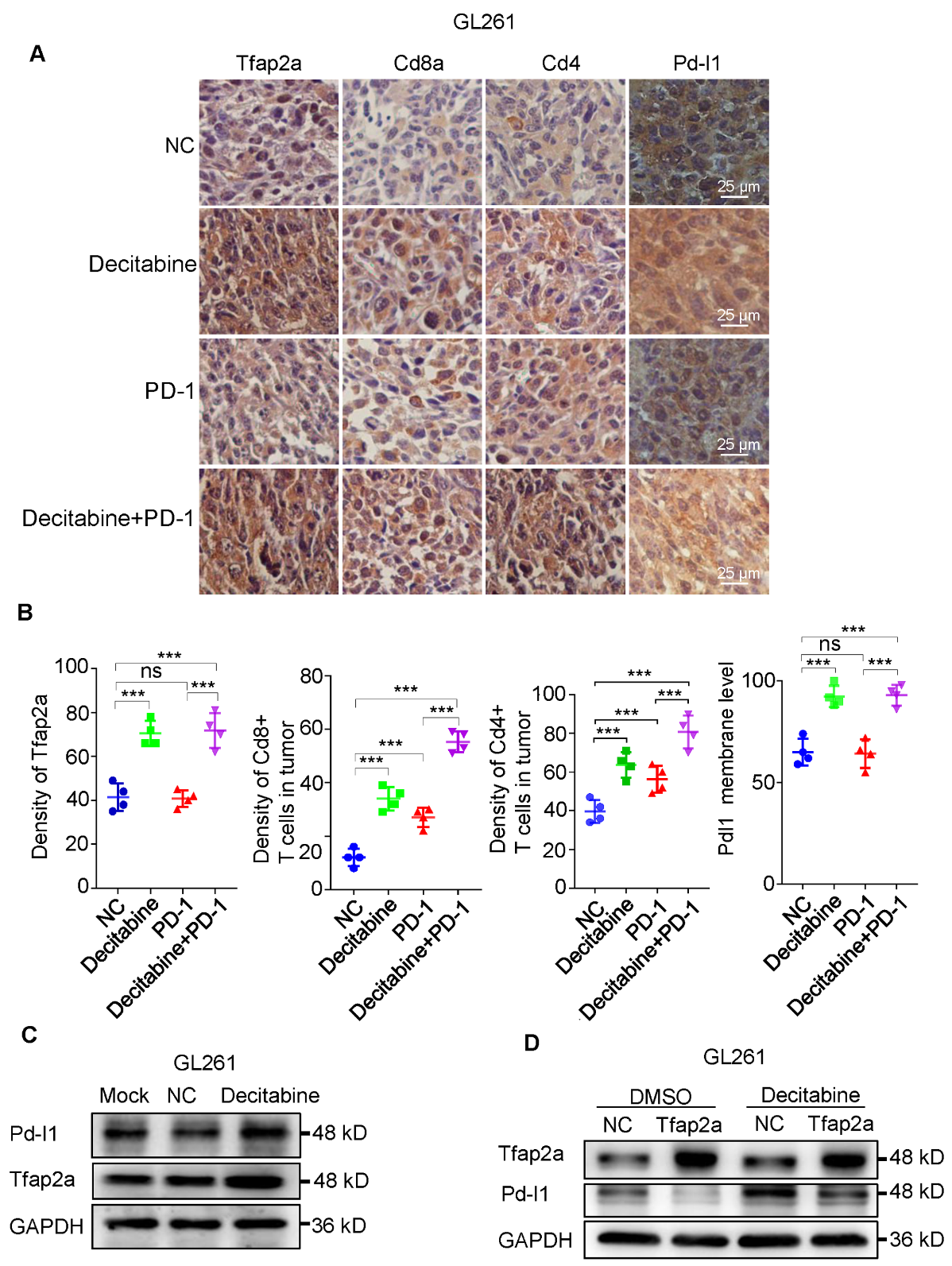


**Supplemental Figure 8 The effects of Decitabine or PD-1 abs on the expression of Tfap2a, Cd8, and Pdl1 in GL261 tumor mice**

A, IHC analysis of the expression of Tfap2a, Cd8, Cd4 and Pdl1 in treated GL261 tumor samples as indicated. B, The corresponding staining scores of target genes in GL261 intracranial tumors from four groups. C, The effects of Decitabine on the expression of Tfap2a and Pdl1 in GL261 cells. D, The effects of Decitabine and Tfap2a on the expression of Pdl1 in GL261 cells.


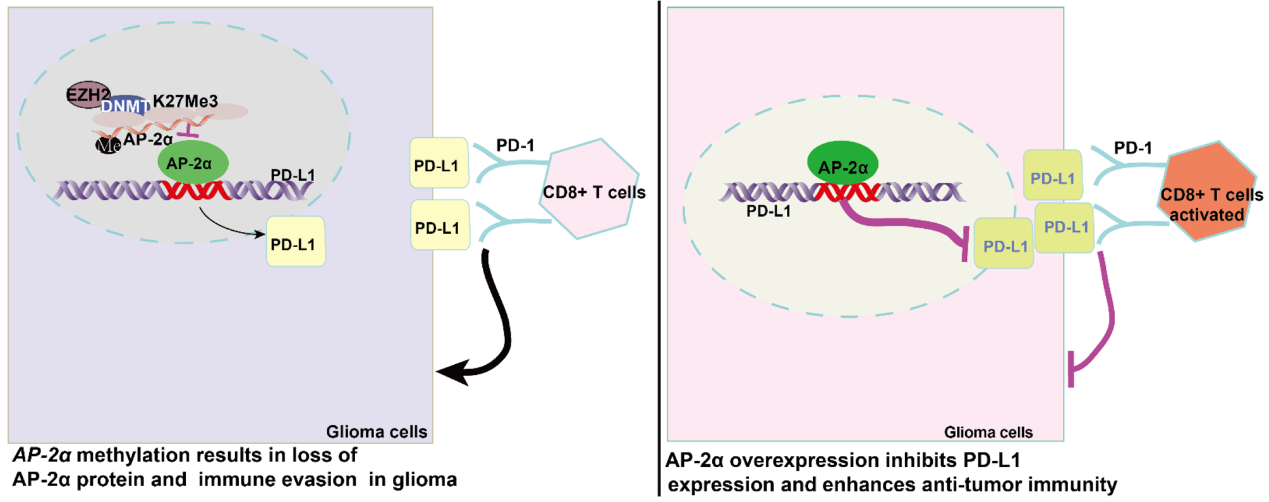


**Supplemental Figure 9 A mechanism model of AP-2α-mediated PD-L1 expression in glioma immune response**

Schematic representation of the mechanisms underlying the recruitment of the epigenetic modification complex to the *AP-2α* promoter, resulting to upregulation of PD-L1 expression and tumor immune evasion. In contrast, improved AP-2α expression could inhibit PD-L1 expression in glioma cells, increase the proliferation and activation of CD8^+^ T cells, and thus augment anti-tumor immunity.

Table S1 Primer pairs in this study

| **Name** | **Sequence (5'- to -3')** | **Purpose** |
| --- | --- | --- |
| AP-2α F | AGGTCAATCTCCCTACACGAG |  |
| AP-2α R | GGAGTAAGGATCTTGCGACTGG | RT-PCR |
| PD-L1 F | TGGCATTTGCTGAACGCATTT |  |
| PD-L1 R | TGCAGCCAGGTCTAATTGTTTT | RT-PCR |
| β-actin F | GCGCGGCTACAGCTTCA |  |
| β-actin R | CTTAATGTCACGCACGATTTCC | RT-PCR |
| GZMB F | CCCTGGGAAAACACTCACACA |  |
| GZMB R | GCACAACTCAATGGTACTGTCG | RT-PCR |
| PPAR-γ F | AGCCTGCGAAAGCCTTTTGGTG |  |
| PPAR-γ R | GGCTTCACATTCAGCAAACCTGG | RT-PCR |
| FoxP3 F | GTGGCCCGGATGTGAGAAG |  |
| FoxP3 R | GGAGCCCTTGTCGGATGATG | RT-PCR |
| Perforin F | CCTTTCCTGCTGGACGAACCAG |  |
| Perforin R | ATGGAGGGCTTTGACGTGTCGT | RT-PCR |
| pGC1-α F | CCAAAGGATGCGCTCTCGTTCA |  |
| pGC1-α R | CGGTGTCTGTAGTGGCTTGACT | RT-PCR |
| TNFα F | GAGGCCAAGCCCTGGTATG |  |
| TNFα R | CGGGCCGATTGATCTCAGC | RT-PCR |
| IFN-γ F | TCGGTAACTGACTTGAATGTCCA |  |
| IFN-γ R | TCGCTTCCCTGTTTTAGCTGC | RT-PCR |
| PD-L1 promoter F | CCGGGTACCCAAGGTGCGTTCAGATGTTG |  |
| PD-L1 promoter R | CGCTCGAGGAAGGCCAGATGCATTGCCT | PCR |
| AP-2α methylation F | **T**TGGAGTGCG**T**T**T**TAGAAG**T**AAGTTCGC |  |
| AP-2α methylation R | A**A**TACCACAATCT**A**CCGCCG**A**CCG | PCR |
| AP-2α unmethylation F | AGG**T**TGGAGTGCG**T**T**T**TAGAAG**T**AAGTT**T**G**T** |  |
| AP-2α unmethylation R | **A**CCA**A**TACCACAATCT**A**CC**A**CC**AA**CC**A** | PCR |
| AP2-promoter F  AP2-promoter R | AAGGTTTTAT**T**AGTT**T**G**T**AGG**T**TGGAG CCTCATTA**A**CATATCAACAATA**A**TCCAATT**A** | PCR(BSP) |
| AP-2α WT1 F | TTTGCTGCCTTGGGCAGAGGTG |  |
| AP-2α WT1 R | CACCTCTGCCCAAGGCAGCAAA | EMSA |
| AP-2α WT2 F | GACCCCGCCTCCGGGCCTGGCG |  |
| AP-2α WT2 R | CGCCAGGCCCGGAGGCGGGGTC | EMSA |
| AP-2α WT3 F | GCGGGTGCCCACGGCCCAGTAT |  |
| AP-2α WT3 R | ATACTGGGCCGTGGGCACCCGC | EMSA |
| AP-2α MUT1 F | TTGCTGaCTTGatCAGAGGTG |  |
| AP-2α MUT1 R | CACCTCTGatCAAGtCAGCAAA | EMSA |
| AP-2α MUT2 F | GACCCCGatTCCtaGCCTGGCG |  |
| AP-2α MUT2 R | CGCCAGGCtaGGAatCGGGGTC | EMSA |
| AP-2α MUT3 F | GCGGGTGatCACatCCCAGTAT |  |
| AP-2α MUT3 R | ATACTGGGatGTGatCACCCGC | EMSA |
| PD-L1 92bp F | TCAGGAAAGTCCAACGCCC | ChIP |
| PD-L1 92bp R | TCAGGAAAGTCCAACGCCC | ChIP |
| PD-L1 115bp F | CAGTTCTGCGCAGCTTCCC | ChIP |
| PD-L1 115bp R  EZH2 siRNA sense  EZH2 siRNA antisense  AP-2α F  AP-2α R  AP-2α siRNA sense  AP-2α siRNA antisense | CGAGCTAGCCAGAGATACTG GAGGUUCAGACGAGCUGAUdTdT AUCAGCUCGUCUGAACCUCdTdT  AAGGTTTTATCAGTTCGCAG  CCTCATTAGCATATCAACAAT  UUUCUCAACCGACAACAUUtt  AAUGUUGUCGGUUGAGAAAtt | ChIP  siRNA  siRNA  ChIP  ChIP  siRNA  siRNA |

**SUPPLENMENTAL REFERENCES**

S1. Yang L, Qiu J, Xiao Y, Hu X, Liu Q, Chen L*, et al.* AP-2beta inhibits hepatocellular carcinoma invasion and metastasis through Slug and Snail to suppress epithelial-mesenchymal transition. Theranostics. 2018; 8**:** 3707-3721.

S2. Huang W, Chen C, Liang Z, Qiu J, Li X, Hu X*, et al.* AP-2α inhibits hepatocellular carcinoma cell growth and migration. Int J Oncol. 2016; 48**:** 1125.

S3. Huang W, Zhong Z, Luo C, Xiao Y, Li L, Zhang X*, et al.* The miR-26a/AP-2alpha/Nanog signaling axis mediates stem cell self-renewal and temozolomide resistance in glioma. Theranostics. 2019; 9**:** 5497-5516.

S4. Wang F, Huang W, Hu X, Chen C, Li X, Qiu J*, et al.* Transcription factor AP-2beta suppresses cervical cancer cell proliferation by promoting the degradation of its interaction partner beta-catenin. Mol Carcinog. 2017; 56**:** 1909-1923.

S5. Ding X, Luo C, Zhou J, Zhong Y, Hu X, Zhou F*, et al.* The interaction of KCTD1 with transcription factor AP-2alpha inhibits its transactivation. J Cell Biochem. 2009; 106**:** 285-295.

S6. Ailan H, Xiangwen X, Daolong R, Lu G, Xiaofeng D, Xi Q*, et al.* Identification of target genes of transcription factor activator protein 2 gamma in breast cancer cells. BMC cancer. 2009; 9**:** 279.

S7. Burr ML, Sparbier CE, Chan YC, Williamson JC, Woods K, Beavis PA*, et al.* CMTM6 maintains the expression of PD-L1 and regulates anti-tumour immunity. Nature. 2017; 549**:** 101-105.

S8. Li Z, Bu J, Zhu X, Zhou H, Ren K, Chu PK*, et al.* Anti-tumor immunity and ferroptosis of hepatocellular carcinoma are enhanced by combined therapy of sorafenib and delivering modified GO-based PD-L1 siRNAs. Biomater Adv. 2022; 136**:** 212761.

S9. Ding X, Luo Y, Zhang X, Zheng H, Yang X, Yang X*, et al.* IL-33-driven ILC2/eosinophil axis in fat is induced by sympathetic tone and suppressed by obesity. J ENDOCRINOL. 2016; 231**:** 35-48.

S10. De Henau O, Rausch M, Winkler D, Campesato LF, Liu C, Cymerman DH*, et al.* Overcoming resistance to checkpoint blockade therapy by targeting PI3Kgamma in myeloid cells. Nature. 2016; 539**:** 443-447.

S11. Douglas DB, Akiyama Y, Carraway H, Belinsky SA, Esteller M, Gabrielson E*, et al.* Hypermethylation of a small CpGuanine-rich region correlates with loss of activator protein-2alpha expression during progression of breast cancer. Cancer Res. 2004; 64**:** 1611-1620.
