# Supplementary material for: Epigenetically modified AP-2α by DNA methyltransferase facilitates glioma immune evasion by upregulating PD-L1 expression
Source: Cell Death Dis. 2023 Jun 17;14(6):365. doi: 10.1038/s41419-023-05878-x (PMC10276877; doi:10.1038/s41419-023-05878-x)

Figure 1G

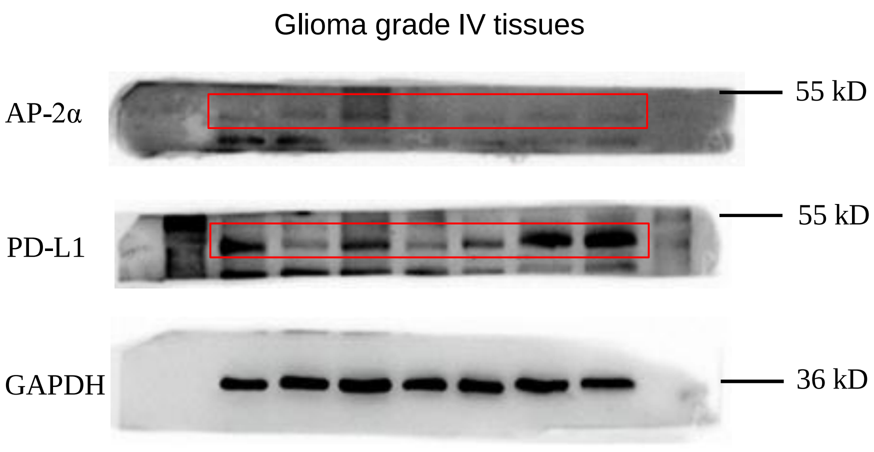

Figure 1H

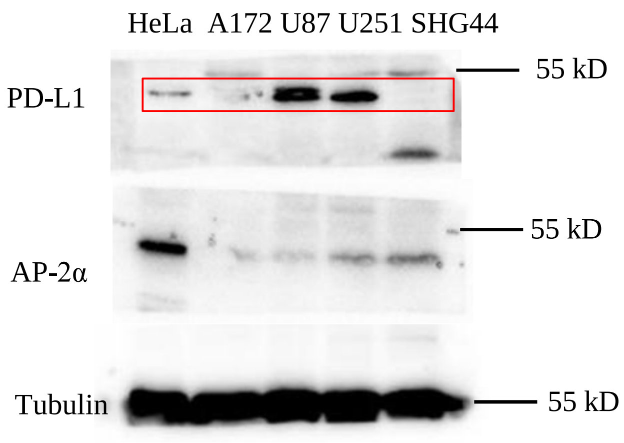

Figure 2C

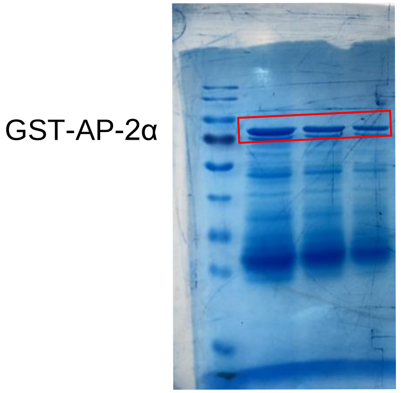

Figure 2D

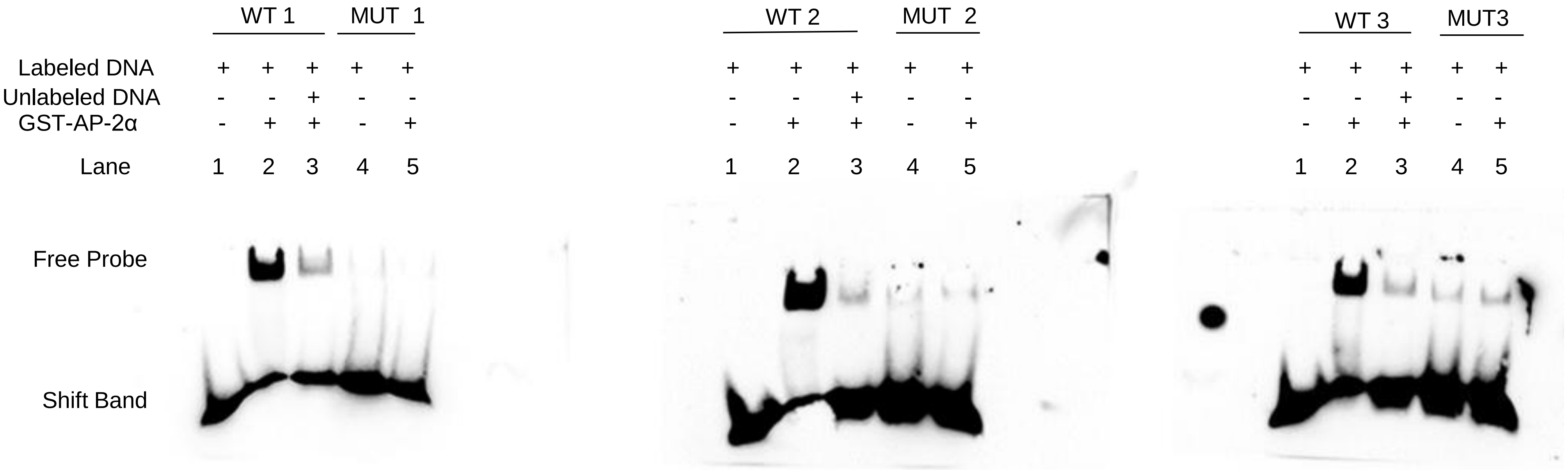

Figure 2E

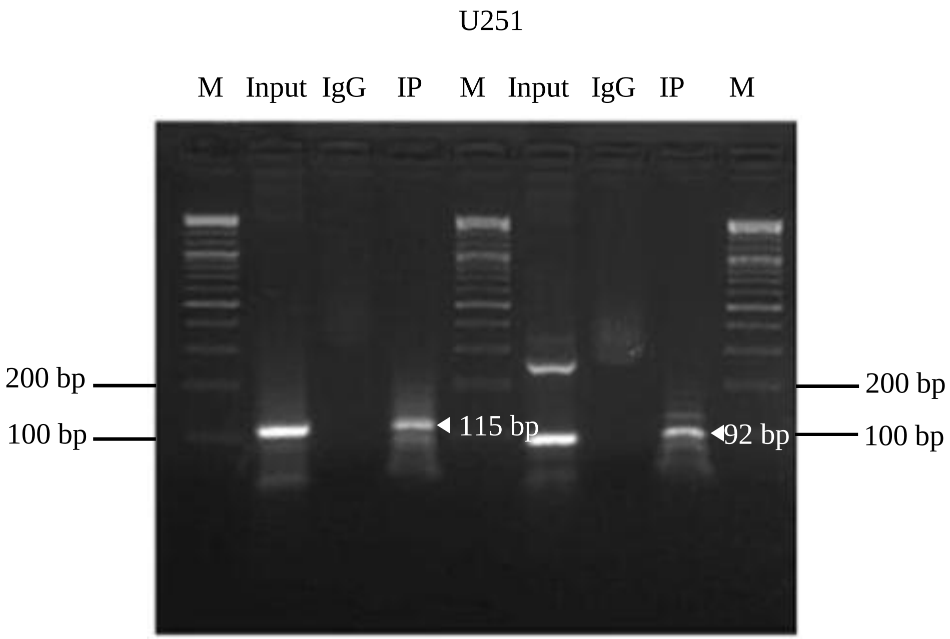
U251
M
Input
IgG
IP
M
Input
IgG
IP
M
200 bp
100 bp
115 bp
92 bp
200 bp
100 bp

Figure 3B

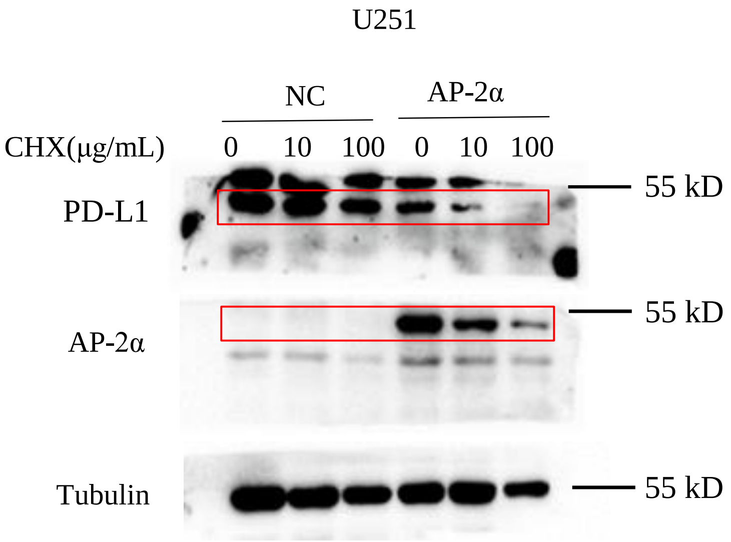

Figure 3C

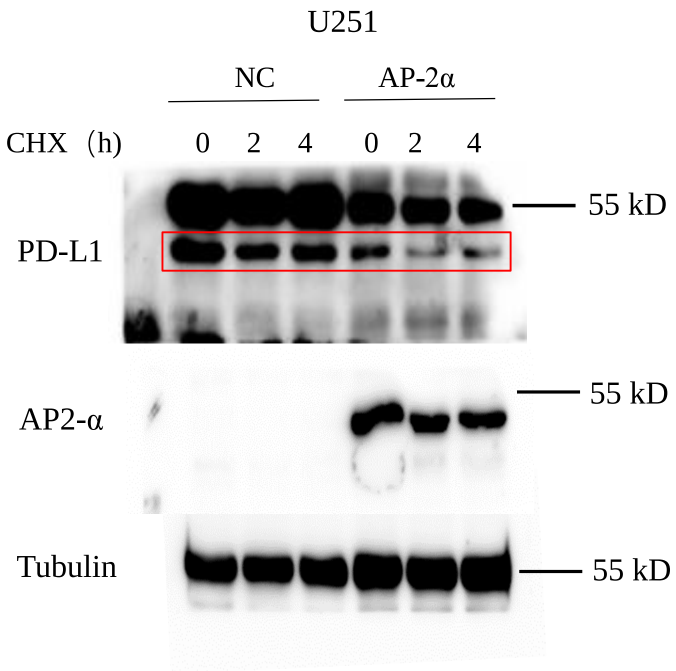

Figure 3D

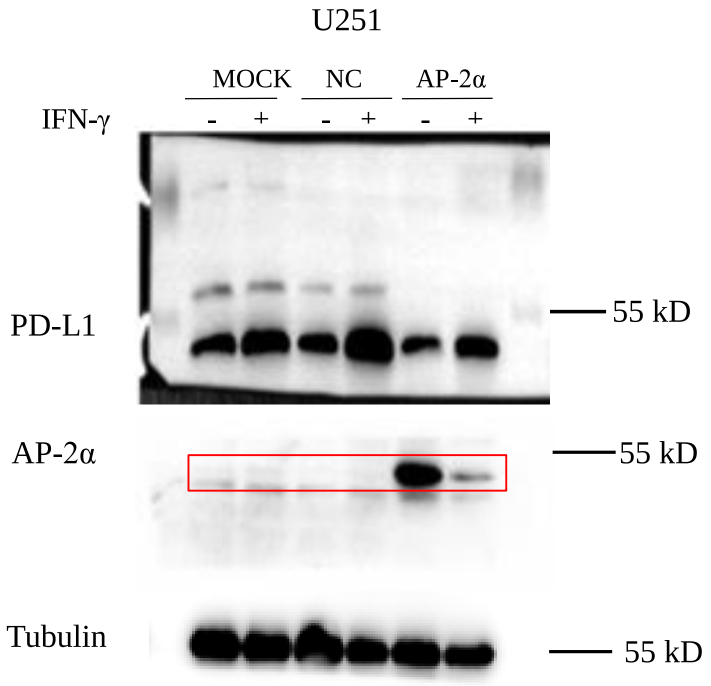

Figure 3E

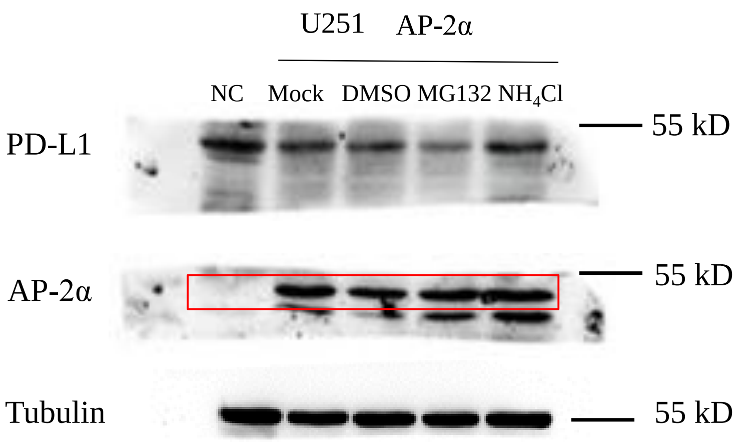

Figure 3G

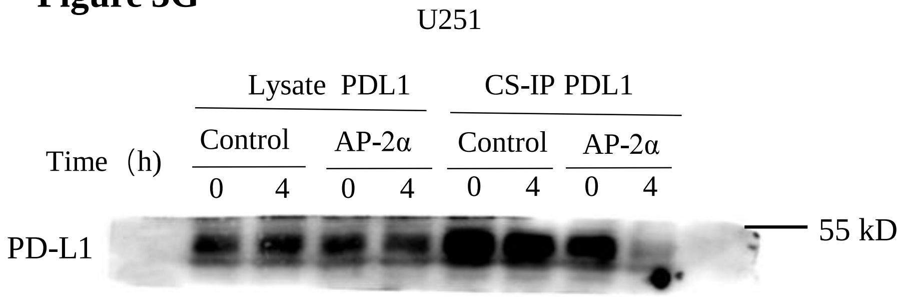

Supplemental Figure 2A

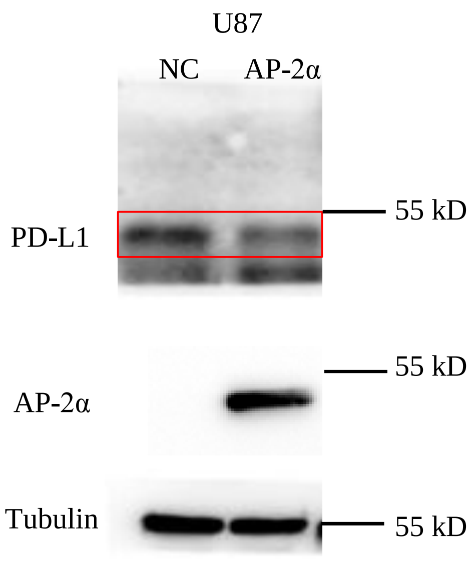

Supplemental Figure 2B

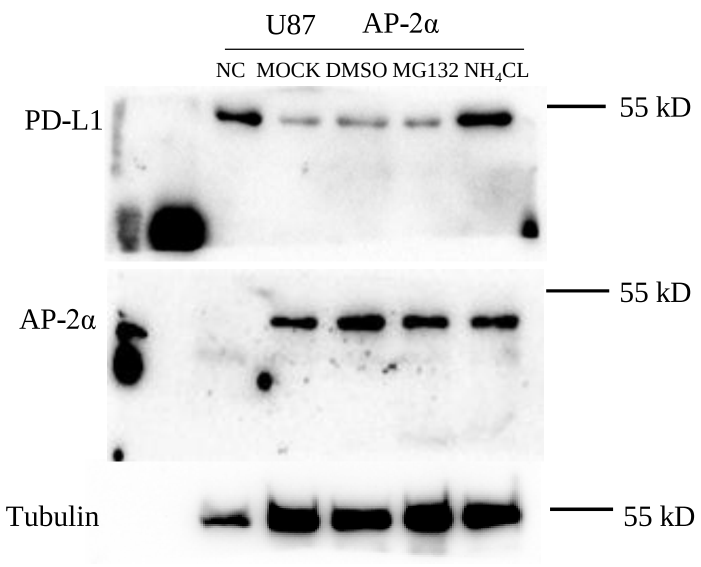

Supplemental Figure 2C

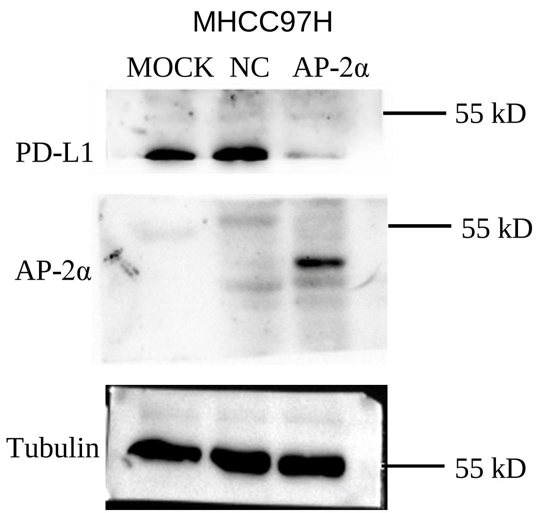

Supplemental Figure 2D

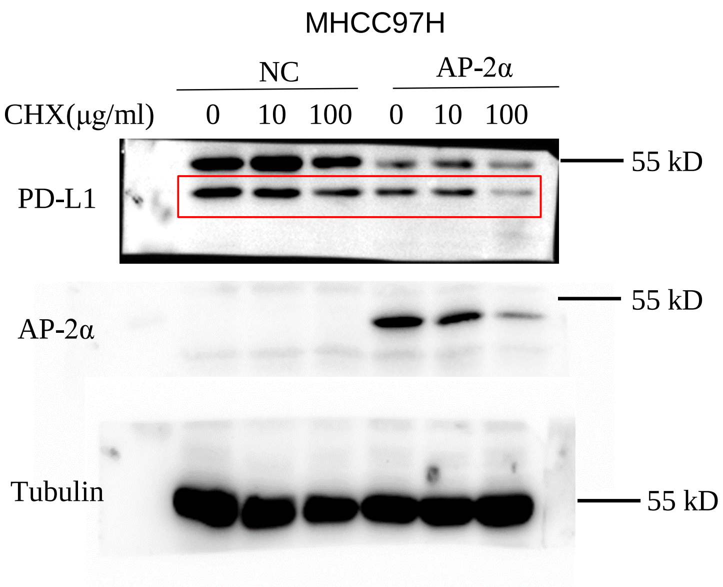

Supplemental Figure 2E

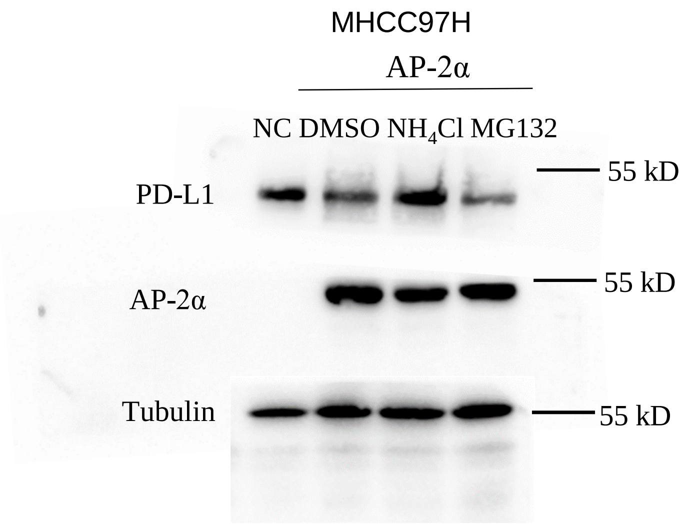

Supplemental Figure 2F

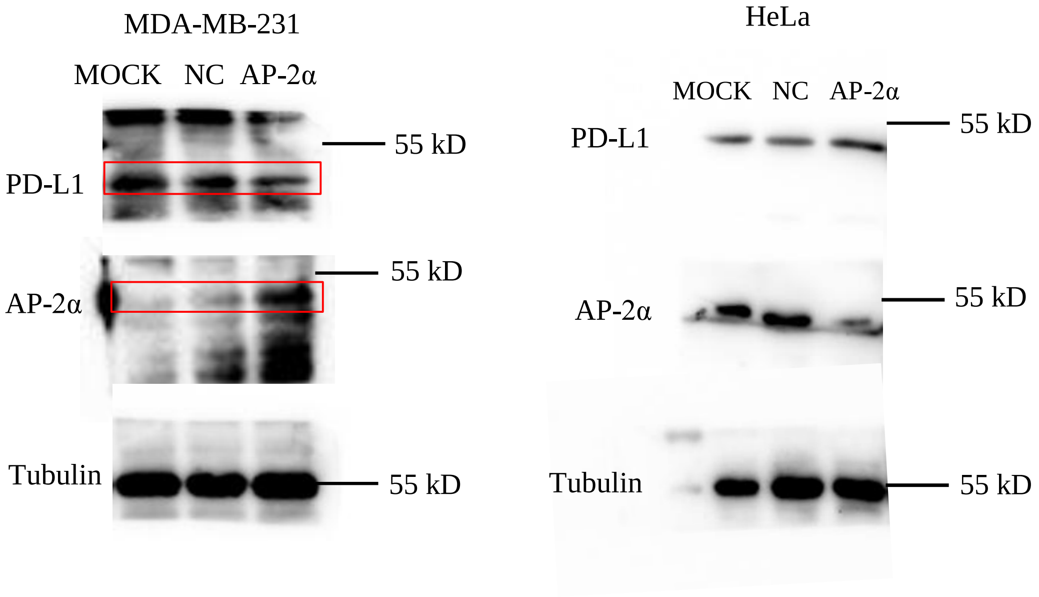

Figure 4H

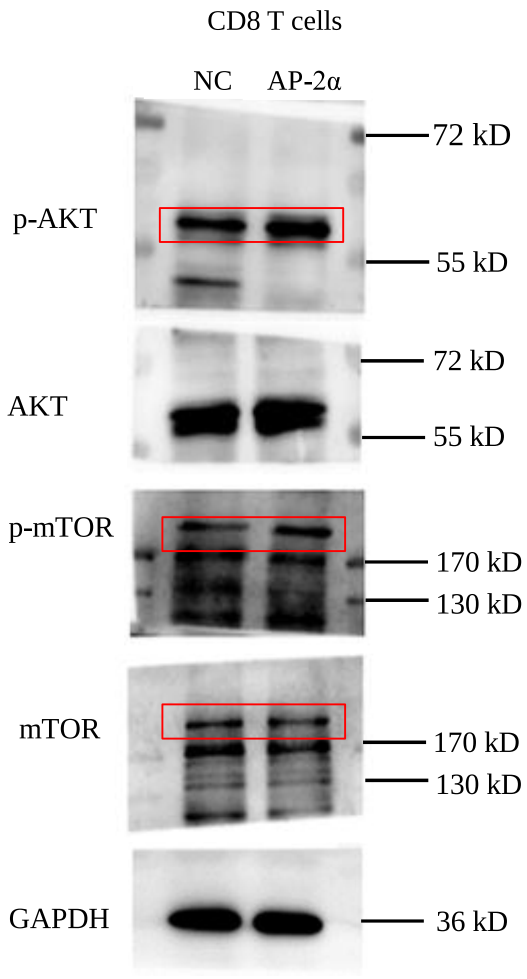
CD8 T cells
NC
AP-2α
p-AKT
AKT
p-mTOR
mTOR
GAPDH
72 kD
55 kD
72 kD
55 kD
170 kD
130 kD
170 kD
130 kD
36 kD

Figure 5B

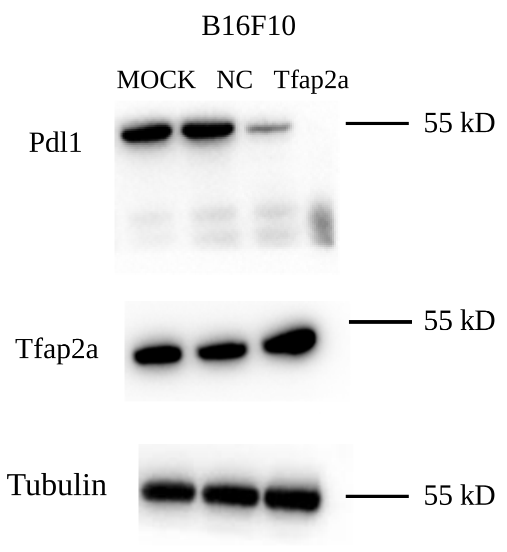

Supplemental Figure 5B

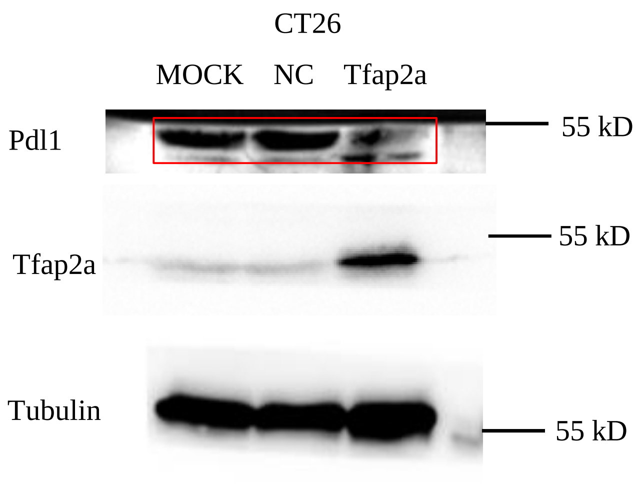

Figure 6C

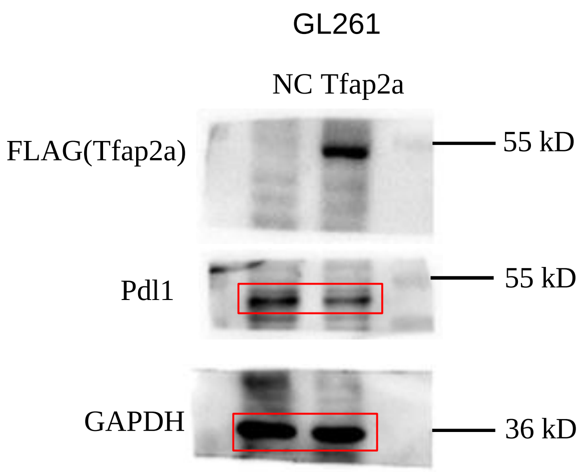

Figure 6D

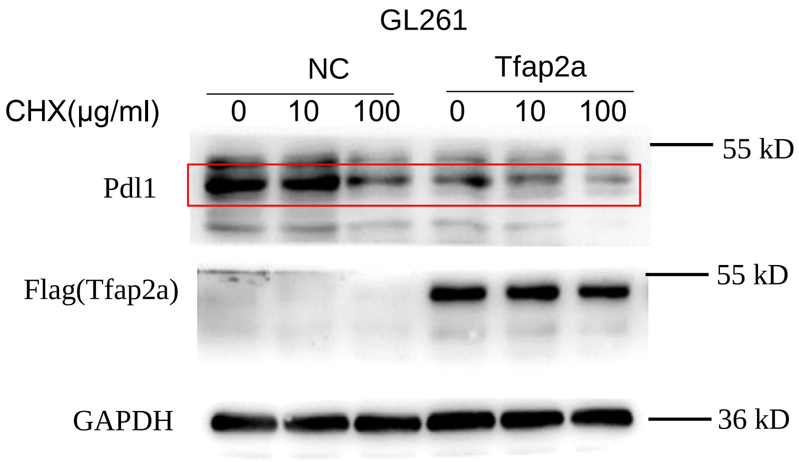

Figure 6E

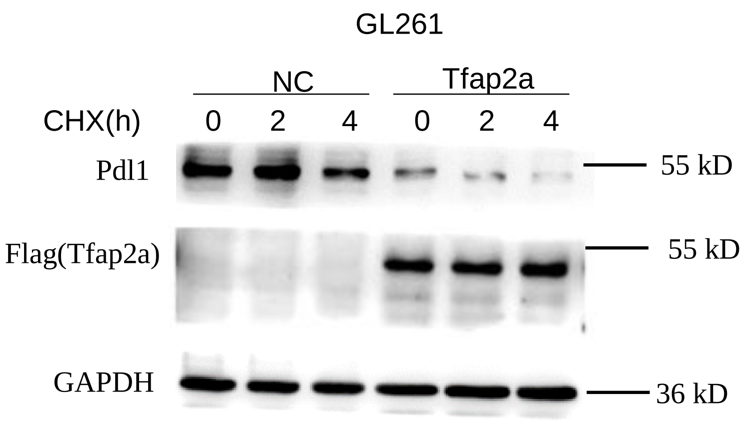

Figure 6F

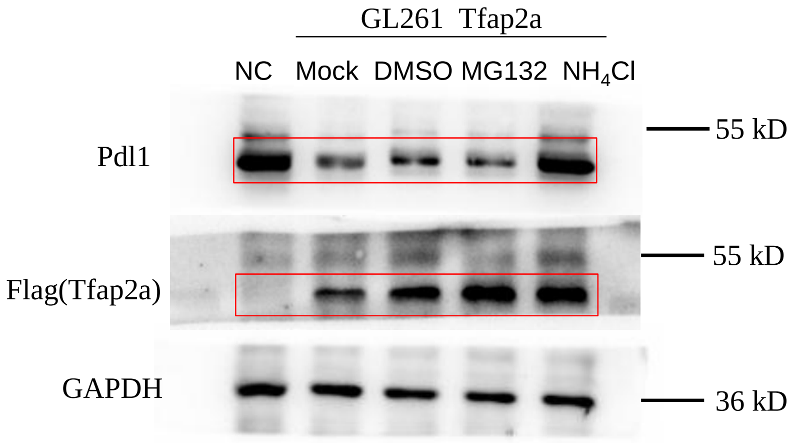

Figure 7B

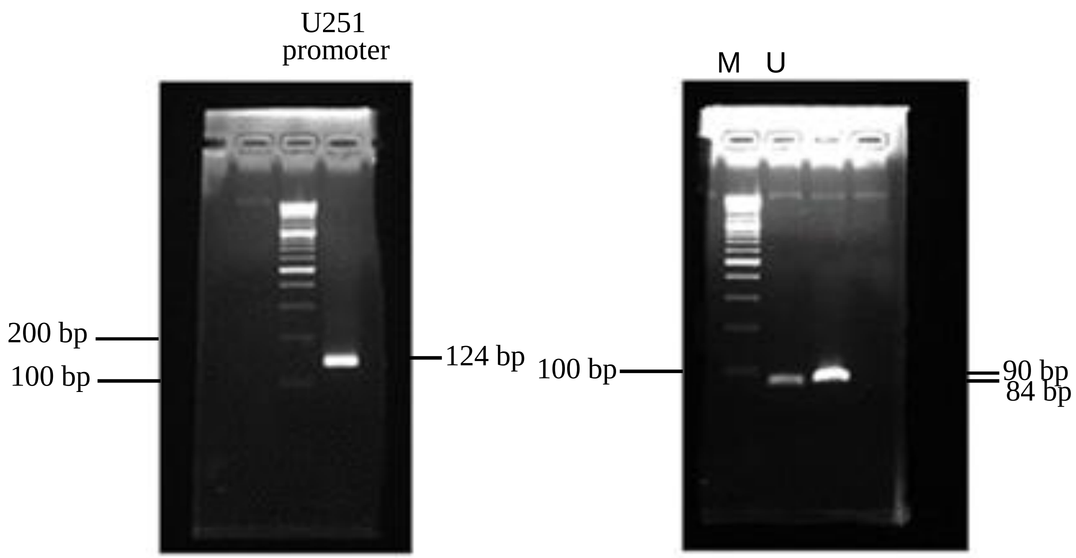

Figure 7C

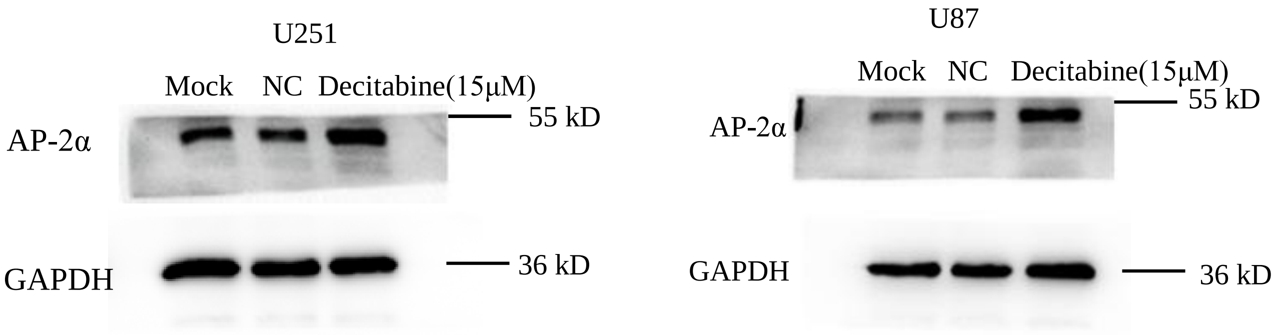

Figure 7F

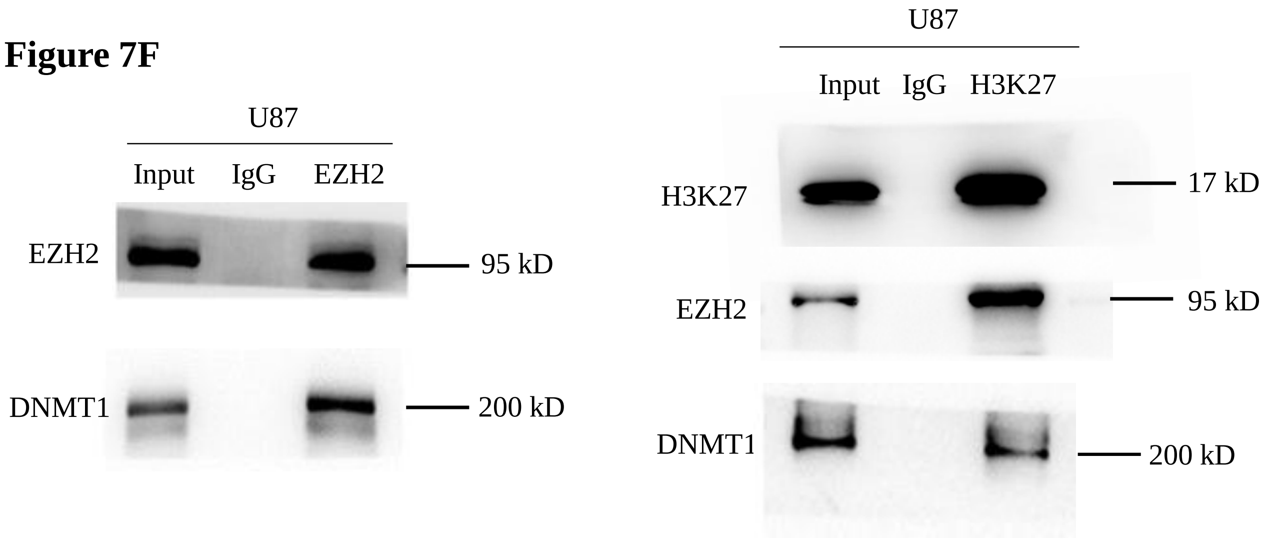

Figure 7G

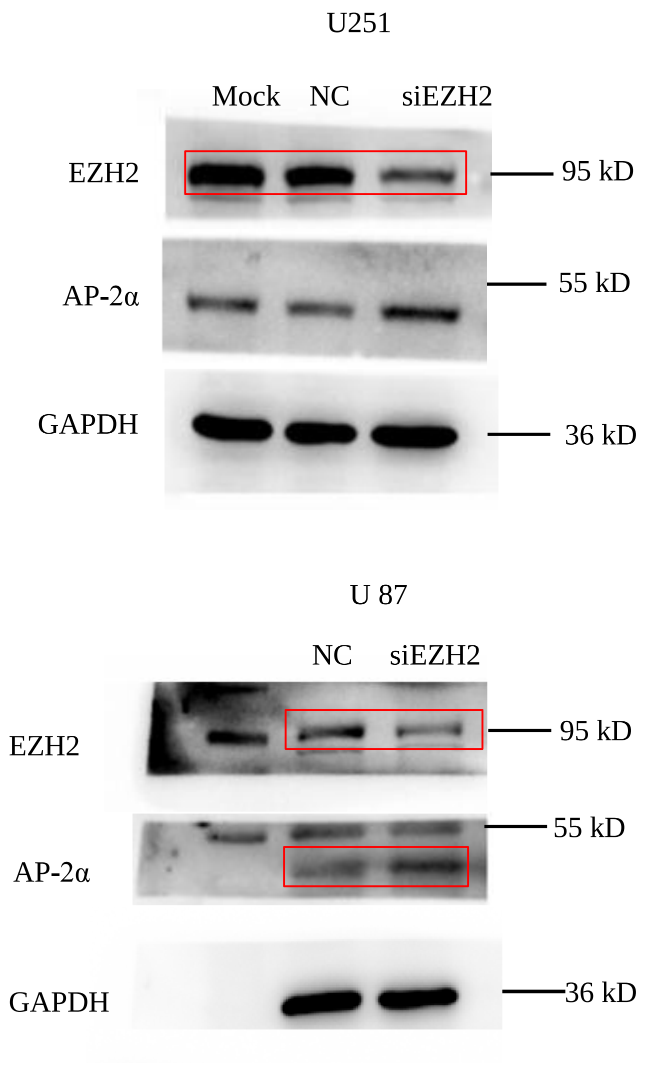

Figure 7H

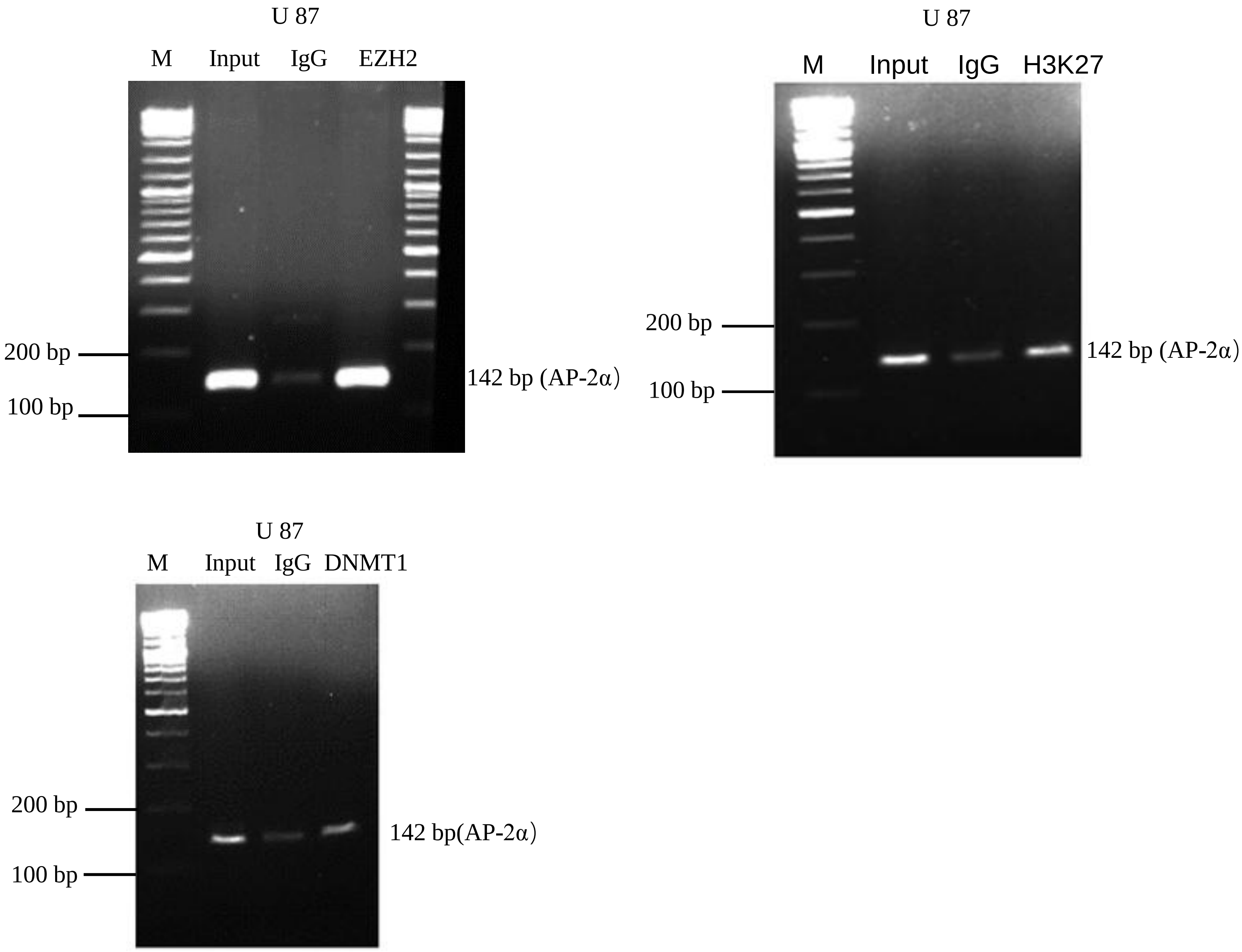

Supplemental Figure 8C

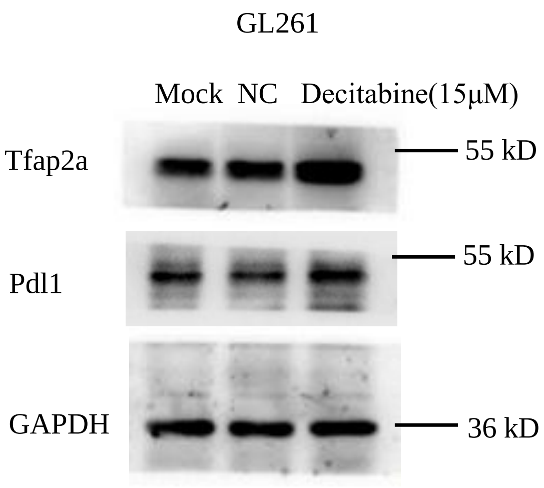

Supplemental Figure 8D

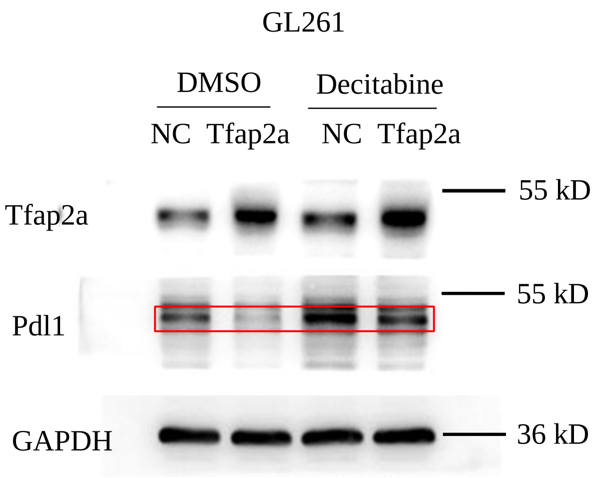

Supplement: Supplementary file 3 — Original Data File [file 41419_2023_5878_MOESM3_ESM.pdf]
